# Comparative pangenomic analysis of predominant human vaginal lactobacilli strains towards population-specific adaptation: understanding the role in sustaining a balanced and healthy vaginal microenvironment

**DOI:** 10.1186/s12864-023-09665-y

**Published:** 2023-09-22

**Authors:** Anupam Bhattacharya, Sushmita Das, Maloyjo Joyraj Bhattacharjee, Ashis K. Mukherjee, Mojibur Rohman Khan

**Affiliations:** 1 Division of Life Sciences, Institute of Advanced Study in Science and Technology, Paschim Boragaon, Guwahati, 781035 Assam India; 2 Department of Biotechnology, Gauhati University, Guwahati, 781014 Assam India

**Keywords:** Gene ontology, GO functional enrichment, Pangenome, Vaginal microenvironment, Vaginal *Lactobacillus*

## Abstract

The vaginal microenvironment of healthy women has a predominance of *Lactobacillus crispatus, L. iners, L. gasseri*, and *L. jensenii*. The genomic repertoire of the strains of each of the species associated with the key attributes thereby regulating a healthy vaginal environment needs a substantial understanding.

We studied all available human strains of the four lactobacilli across different countries, isolated from vaginal and urinal sources through phylogenetic and pangenomic approaches. The findings showed that *L. iners* has the highest retention of core genes, and *L. crispatus* has more gene gain in the evolutionary stratum. Interestingly, *L. gasseri* and *L. jensenii* demonstrated major population-specific gene-cluster gain/loss associated with bacteriocin synthesis, iron chelating, adherence, zinc and ATP binding proteins, and hydrolase activity. Gene ontology enrichment analysis revealed that *L. crispatus* strains showed greater enrichment of functions related to plasma membrane integrity, biosurfactant, hydrogen peroxide synthesis, and iron sequestration as an ancestral derived core function, while bacteriocin and organic acid biosynthesis are strain-specific accessory enriched functions. *L. jensenii* showed greater enrichment of functions related to adherence, aggregation, and exopolysaccharide synthesis. Notably, the key functionalities are heterogeneously enriched in some specific strains of *L. iners* and *L. gasseri*.

This study shed light on the genomic features and their variability that provides advantageous attributes to predominant vaginal *Lactobacillus* species maintaining vaginal homeostasis. These findings evoke the need to consider region-specific candidate strains of *Lactobacillus* to formulate prophylactic measures against vaginal dysbiosis for women’s health.

**Supplementary Information:**

The online version contains supplementary material available at 10.1186/s12864-023-09665-y.

## Significance

The vaginal microenvironment is dominated by *Lactobacillus crispatus, L. iners, L. gasseri, and L. jensenii*, which acts as a gatekeeper in the vaginal ecosystem retarding the growth of pathogens. The species co-habits in the vaginal microenvironment separating their niches, and bearing genomic repertoire for advantageous adaptive attributes in the vaginal microenvironment such as plasma membrane plasticity, adherence, aggregation, and cell surface active molecules such as exopolysaccharides and biosurfactants.

This study performed pangenomic analysis of predominant human vaginal lactobacilli strains in population-specific adaptation. More importantly, the study shed light on genomic features and their variability that provides advantageous attributes to predominant vaginal *Lactobacillus* species maintaining vaginal homeostasis. We found that few *Lactobacillus* species have region-specific strains with gene cluster gain/loss for tentatively adapting to the human population with diverse genomic repertoire. These findings reveal the genomic evolution of strains of predominant human vaginal lactobacilli for population-specific adaptation and evoke the need to consider region-specific candidate strains of *Lactobacillus* to formulate prophylactic measures against vaginal dysbiosis for women’s health.

**Supplementary Information:**

The online version contains supplementary material available at 10.1186/s12864-023-09665-y.

## Introduction

Vaginal micro ecosystems undergo a continuous shift throughout a woman’s life cycle. The colonization of microbes in the vaginal microenvironment is shaped by factors such as age, hormonal flux, ethnicity, sexual behaviour, feminine hygiene, medication, and lifestyle habits [1]. However, mounting pieces of evidence suggest that a healthy vaginal microenvironment is mainly dominated by *Lactobacillus* which act as vaginal gatekeepers retarding the growth of pathogens [2–4]. Notably, vaginal microbiomes across different populations around the globe share four predominant clusters of lactobacilli viz. *L. crispatus, L. iners, L. gasseri*, and *L. jensenii* [1, 5, 6]. This evokes the need to understand the genomic repertoire of these four species for their widespread colonization in maintaining vaginal homeostasis.

The lactobacilli of the vagina bear many unique attributes, which help in their dominance and to better compete with pathogens in the vaginal microenvironment [7]. Most vital bacterial cell functions are attributed to the plasma membrane to adapt to a wide range of environments such as control on the biophysical property of the membrane, by synthesizing its major constituents like protein, lipids, and phospholipids [8]. Adherence, which is guided by the cell surface factors of the host and the microbe, is another important attribute of vaginal lactobacilli and they outperform pathogenic microbes for adherence in vaginal epithelial cells [9]. Similarly, self-aggregation is another important characteristic of vaginal lactobacilli [10, 11] which allows them to form biofilm by co-aggregation in the vaginal environment. Nevertheless, the lactobacilli also aggregate with the pathogen for their lysis either by synthesis of bacteriocins, hydrogen peroxide, etc., or by initiating NF-kB signal transduction pathway releasing cytokines [12–14]. Furthermore, cell surface active molecules (SAMs) of vaginal lactobacilli such as exopolysaccharides and biosurfactants (glycolipids, lipopeptides, lipopolysaccharide), are pivotal attributes for dominance in the vaginal microenvironment [15, 16]. Thus, it is very important to understand the genomic signatures of these important attributes, and their changing landscape across different species/strains to shed light on the genomic basis of adaptation of a particular *Lactobacillus* species/strain in the vaginal microenvironment of a particular population. Therefore, for an in-depth understanding of their predominance in the vaginal microenvironment, we performed a comparative genomic analysis of the four *Lactobacillus* sp. based on their pangenome.

Considering the key attributes of lactobacilli that exert sustained dominance on the vaginal mucosa, previous studies primarily focussed on genomic loci regulating host and body-site specific adaptation [14], bacteriocin production [17], carbohydrate-transport [18], probiotic properties [19], glycosylation and glycogen degradation [20] mostly in *L. crispatus*, and phosphate acquisition mechanism unique in *L. iners* [21]. Notably, such studies are very limited for the other vaginal *Lactobacillus* species although the microbial community state types and dominance of lactobacilli vary across populations. More importantly, each of the key attributes involves a cascade of biological processes but no study has explained the changing landscape of the biological processes representing the key attributes in the four *Lactobacillus* sp. across populations. Furthermore, the studies have not related the pangenome with phylogeny to critically discuss episodes of gene gain and loss during evolution.

The vivid approaches adopted for this study were phylogenomic, comparative genomic, and pangenomic reconstruction analyses. The gene ontology library of microbes has been used to identify the genes involved in key attributes and their variability to gain and loss has been enumerated using the comparative genomic and pangenomic landscape. The phylogeny served the purpose to trace sequential characteristic changes across diverse populations. This study aims to shed light on (i) the genomic features and their variability that provides advantageous attributes to predominant vaginal *Lactobacillus* species maintaining vaginal homeostasis, (ii) the reason for the co-habiting attributes of lactobacilli partitioning and sharing the vaginal niche to bring out combined protective functionality to the host, and (iii) the need to consider region-specific candidate strains of *Lactobacillus* to formulate an effective population-specific vaginal probiotic as a prophylactic measure against vaginal dysbiosis for women’s health.

## Results

### Dataset characteristics

In this study, the whole genome sequences (WGS) of *L. crispatus, L. iners, L. jensenii*, and *L. gasseri* from vaginal origin were downloaded from the NCBI database. The dataset includes strains of each of the four species from different populations of the world (Table 1, Additional file 1: Table S1 sheets 1–4). For *L. crispatus*, we have collected 82 WGS that covered populations from the USA (30), Belgium (2), Brazil (4), China (1), France (2), India (3), Italy (9), the Netherlands (28), Russia (1), South Korea (1), and Thailand (1). Similarly, for *L. iners* we have collected 25 sequences that covered the populations from India (1), Korea (1), Sweden (2) and the USA (21). For *L. gasseri*, we have collected 21 WGS that covered the populations from Italy (1), South Korea (2), and the USA (18) For *L. jensenii*, we have collected 32 WGS from China (1), South Korea (1), Thailand (1) and USA (29). The average whole genome size for *L. crispatus, L. iners, L. jensenii*, and *L. gasseri* is 2.03 Mb, 1.36 Mb, 1.59 Mb, and 1.87 Mb, respectively and most of them are in the form of single or multiple scaffolds.

**Table 1:** Country-wise distribution of lactobacilli genome datasets for pangenome analysis. The data were downloaded from NCBI.

*Lactobacillus crispatus*		*Lactobacillus gasseri*		*Lactobacillus iners*		*Lactobacillus jensenii*	
Country	Processed genomes	Country	Processed genomes	Country	Processed genomes	Country	Processed genomes
Belgium	2	Italy	1	India	1	China	1
Brazil	4	South Korea	2	Korea	1	South Korea	1
China	1	USA	18	Sweden	2	Thailand	1
France	2			USA	21	USA	29
India	3						
Italy	9						
Netherland	28						
Russia	1						
South Korea	1						
Thailand	1						
USA	30						

### Pangenome profile and phylogeny of the bacterial strains

**Fig. 1:**
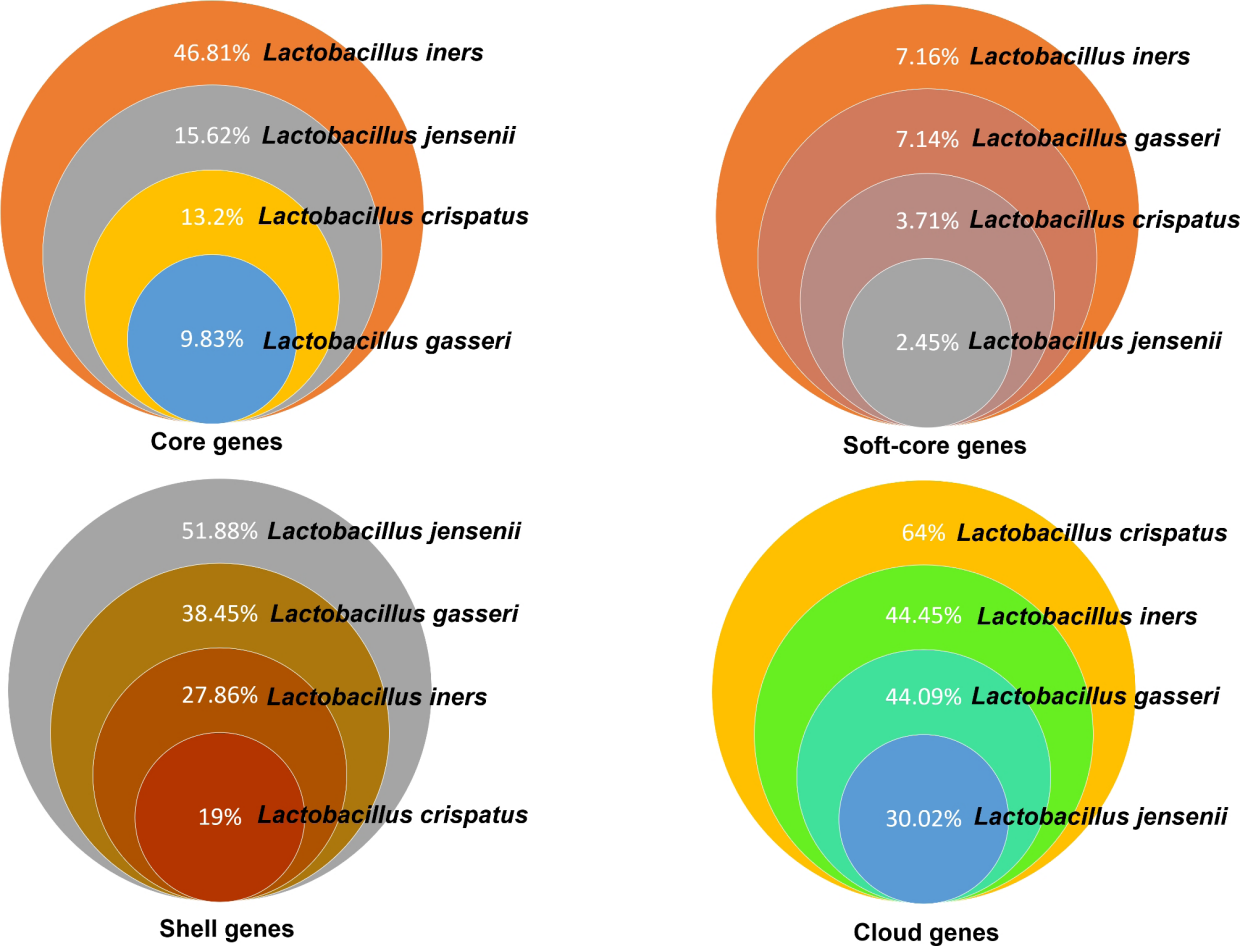
Distribution of the percentage of core, soft-core, shell, and cloud genes among the predominant vaginal lactobacilli. Among the total genes in each of the species, *L. iners* retained a relatively high percentage of core-genes and *L. gasseri* retained the least. *L. crispatus* showed a relatively high percentage of cloud genes which depicts the evolutionary diversification of its strains distributed across different geographic regions

Prokka’s pipeline critically annotated the bacterial genes present in the genomes. Subsequently, Roary’s pipeline compared the annotated genes among the genome sequences of a species. These steps provided a pangenome profile for *L. crispatus, L. iners, L. jensenii*, and *L. gasseri* as shown in the Additional file 1: Table S1 sheet 5–8. A schematic representation of the hierarchical percentage presence of core, soft-core, shell, and cloud genes among *L. crispatus, L. iners, L. jensenii*, and *L. gasseri* is shown in Fig. 1. This data reveals that, among the four dominant *Lactobacillus* species inhabiting the vaginal microenvironment, the diversification of their strains is associated with relatively high percentage retention of core genes in *L. iners* and least in *L. gasseri*. Notably, *L. crispatus* has the highest percentage of cloud genes which reveals that during its evolutionary continuum, new genes were added to the genome. Among the others, *L. jensenii* and *L. iners* have the highest and lowest percentage of soft-core genes, respectively. Whereas, *L. crispatus* and *L. jensenii* have the highest and lowest percentage of shell genes, respectively.

**Fig. 2:**
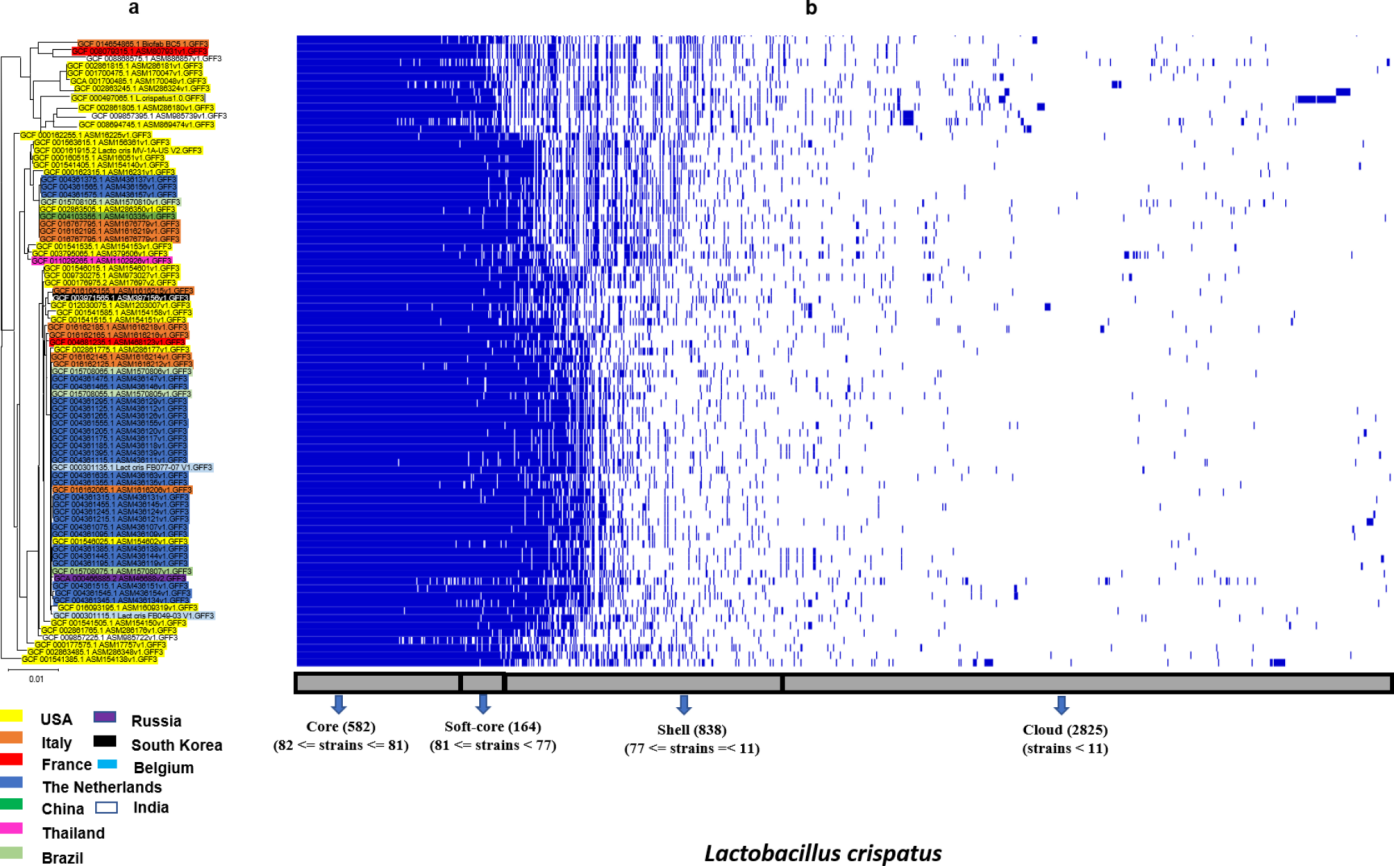
(**a**) Maximum likelihood phylogenetic tree, and (**b**) corresponding pangenome profile based on whole genome sequences of *L. crispatus*. The phylogeny revealed that for *L. cripatus*, the sequences from eleven countries assembled both cohesively (country-wise) and also as distinct clade. In many cases sequences from two different countries clustered together. The pangenome corresponding to the phylogenetic arrangement of the sequences showed no cases of clade-associated gene cluster gain/loss. The sequences with a colour code represents the sequences from a particular country

**Fig. 3:**
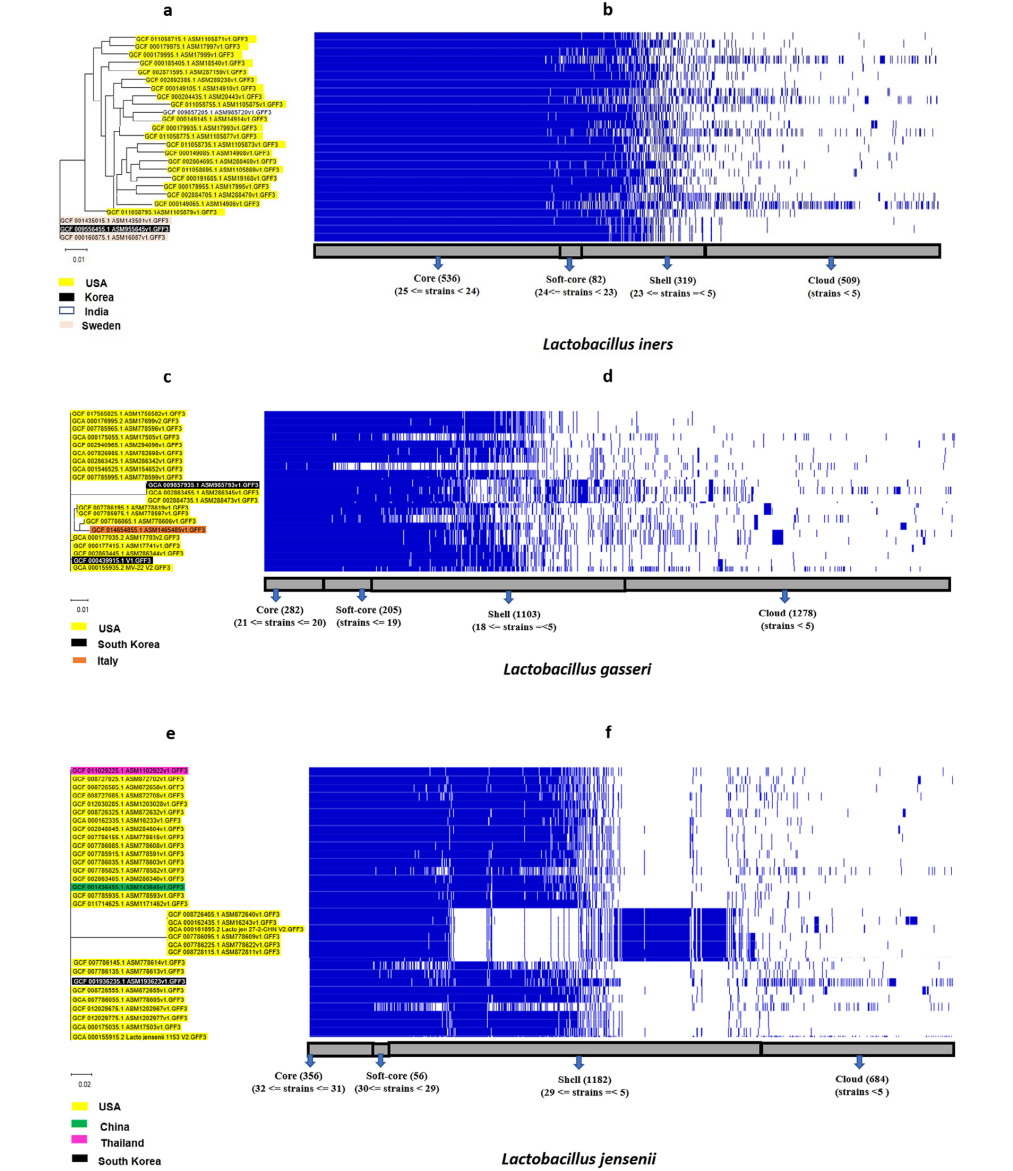
(**a**, **c**, & **e**) Maximum likelihood phylogenetic tree, and 3 (**b**, **d**, & **f**) corresponding pangenome profile based on whole genome sequences of *L. iners, L. gasseri*, & *L. jensenii*. The phylogeny of *L. iners* is prepared by taking available representative sequences from four countries. The cladding of the sequences is mostly country-wise. While *L. crispatus* sequences from South Korea clustered within a USA clade (Fig. 2a), *L. iners* from Korea clustered with Sweden clade. The pangenome of *L. iners* showed a higher proportion of core-genes, which reveals high retention of ancestral genes. Sequences from three countries represent the phylogeny of *L. gasseri*. The phylogeny of *L. gasseri* shows differential cladding within the sequences from the USA and South Korea. While, the sequences from the other country clustered within separate clades of the USA. The pangenome profile reveals gene-cluster gain/loss episodes in a clade represented by three sequences from South Korea and two sequences from USA. Sequences from four countries represented the phylogeny of *L. jensenii*. The phylogeny of *L. jensenii* showed three distinct cladding of the USA strains while, the representative sequences from other countries clustered separately within the USA clades. The pangenome profile showed gene-cluster gain/loss episodes in one USA clade comprising six sequences from the USA

Since the core genes represent the consensus genomic region of all the strains of a species, the core gene alignment file from Roary’s output was used to deduce an ML tree for each of the four species. Figures. 2a, 3a, c, and e show the ML tree of *L. crispatus, L. iners, L. jensenii*, and *L. gasseri* strains, and Figs. 2b and 3b, d, and f, show their corresponding pangenome profile. Initially, we did the phylogenetic reconstruction of the strains for each of the species, and subsequently tried to understand the episodes of gene cluster gain/loss (from the pangenome profile) relating to the phylogenetic clades in the succeeding section.

It is evident from the ML tree that, for *L. crispatus* (Fig. 2a), a few strains from the USA represents the basal clade, and it is successively followed by a heterogeneous assemblage of USA strains in several small distinct clades. The strains from the Netherlands formed five sister clades and one distinct clade. The strains from Italy formed five clades and that from France formed a single clade. The single strain from South Korea clustered as a sister clade with one of the clades of Italy.

Among the three Indian strains, one clustered at the basal, and the two other strains formed two distinct clades close to a USA clade. The single strains from China clustered within an Italian clade and one strain from Thailand clustered within a USA clade. The Brazilian strains and a Belgium strain clustered within the Netherlands clade, while another strain from Belgium clustered basal with few USA strains. For *L. iners* (Fig. 3a), the Sweden and the South Korean strains formed the basal clade, and all USA and a single strain of India clustered within a clade (Fig, 3c).

In *L. gasseri*(Fig. 3b), the USA strains are heterogeneously assembled into distinct clades. The strain from Italy and South Korea clustered within separate distinct clades of the USA strains. In *L. jensenii* (Fig. 3e), the USA strains formed three distinct clades, and the other strains from China, Thailand, and South Korea clustered within either of the three clades of USA strains.

### Gain-and-loss of major gene clusters in the predominant *Lactobacillus* species of vaginal microenvironment

While we assessed the pangenome profile of the four *Lactobacillus* species, we anticipate that any significant gain/loss of gene cluster associated with a particular clade (Figs. 2a and 3a and c, & e) will be concentrated on the ‘shell’ and the ‘cloud’ region of the pangenome (Figs. 2b, 3b, d, and f). *L. crispatus* (Fig. 2b) and *L. iners* (Fig. 3b) have no significant gene cluster gain/loss that may be associated with a particular clade or sequences from a particular region.

Notably, in *L. gasseri* (Fig. 3d), a clade having two strains each from the USA and one strain from South Korea showed a significant gene cluster loss/gain (Additional file 1: Table S1 sheet 9) compared to the strains in two other clades, one having strains only from the USA and the other having strains from the USA, Italy, and one strain from South Korea. Similarly, in *L. jensenii* (Fig. 3f), a clade represented by six USA strains showed significant loss/gain of the gene cluster (Additional file 1: Table S1 sheet 10) compared to the other clades represented by strains from the USA, China, Thailand, and South Korea. The gain/loss of functions related to the gain/loss of gene clusters is explained in the succeeding section.

### Functional enrichment of pangenome of different *Lactobacillus* species and gain-and-loss of key functions

The pangenome of each of the four species (Figs. 2b, 3b, d, and f) have been subdivided into core, soft-core, shell, and cloud genes based on the percentage of the strains of a species having the genes. As such, the core, soft-core, shell, and cloud genes represent the genes found among 98 to 100%, 94 to 97%, 14 to 93%, and 1 to 13% strains, respectively. Here, we have considered the cluster of the genes under core, soft-core, shell, and cloud as four groups of Gene Ontology (GO) and detailed results of significant functional enrichment analysis of the GOs using ClueGo, EnrichR, and ShinyGO search platform is given in the Additional file 2: Figure S1 and S2 for *L. crispatus* and *L. iners* respectively and the Additional file 2: Figure S3a and b, and S4 for *L. gasseri* and *L. jensenii* respectively.

**Fig. 4:**
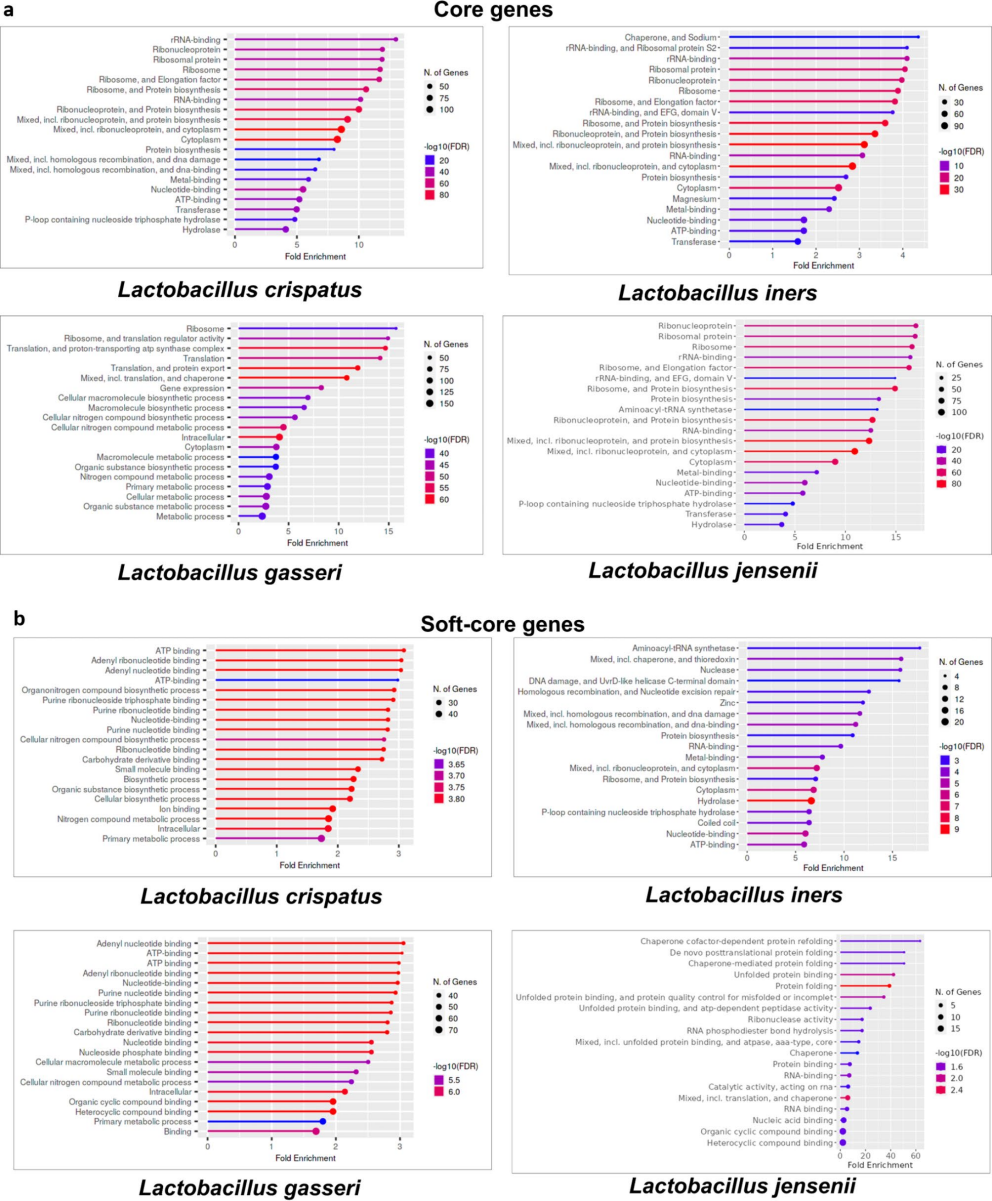
Functional enrichment (FDR cutoff 0.05) of the (**a**) core and (**b**) soft-core gene cluster of the predominant vaginal lactobacilli

The core and the soft-core represent the genes primarily present in all the strains of the respective species. Figure 4a and b show the enriched GO terms of the core and soft-core genes of the four predominant species of lactobacilli. The figures depict that the biological functions such as protein biosynthesis, DNA damage and repair, binding activities related to nucleotide, ATP, ribonucleotide, purines, and other small molecules and ions, enzymatic activities such as hydrolase and transferase, cellular and organic biosynthetic process, and some primary intracellular metabolic processes are the conserved functions in all the strains of *L. crispatus*. Therefore, these are the enriched GO terms based on the pangenomic core and soft-core genes of the species. These biological functions are also enriched GO terms based on the core and soft-core genes of *L. iners, L. gasseri*, and *L. jensenii.* Besides, there are other shared functions among the *Lactobacillus* species such as chaperone-supported protein folding (*L. iners* and *L. jensenii*), organonitrogen compound biosynthesis (*L. gasseri* and *L. crispatus*), carbohydrate derivative binding (*L. crispatus* and *L. gasseri*), and magnesium, zinc and growth-factor binding (*L. iners* and *L. jensenii*).

**Fig. 5:**
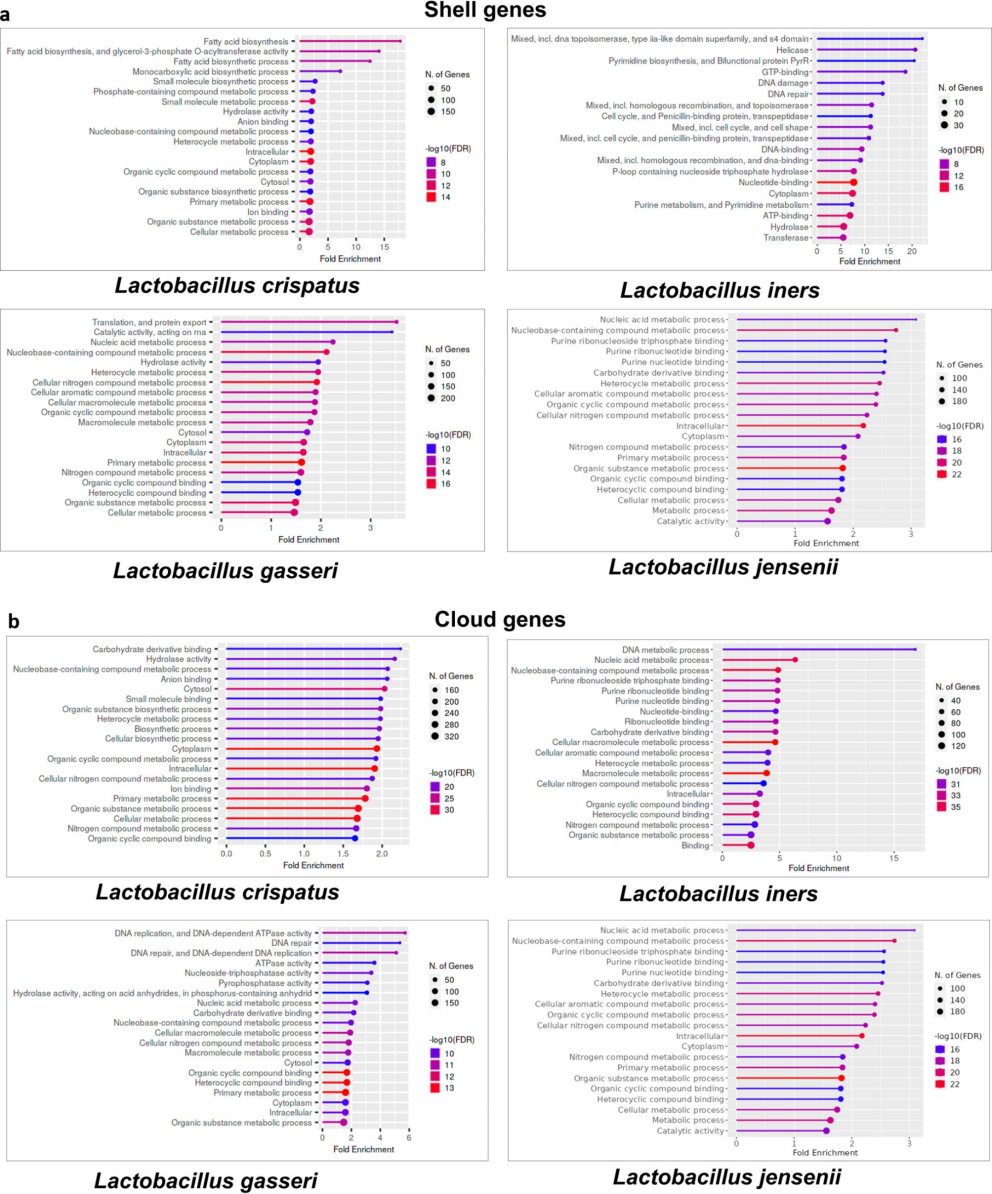
Functional enrichment (FDR cutoff 0.05) of the (**a**) shell and (**b**) cloud gene cluster of the predominant vaginal lactobacilli

The shell and the cloud mostly represent the genes gained/lost among some of the strains of a species during evolution or adaptation to a particular environment. Figure 5a and b show the enriched GO terms of the shell and cloud genes of the four predominant species of lactobacilli. The enriched GO terms based on the shell-cloud genes of *L. crispatus* relate to fatty acid biosynthesis, carboxylic acid biosynthesis, phosphate-linked compound metabolic process, and organic substance metabolic process. It is noteworthy to mention that some of the core and soft-core gene-based functions such as heterocyclic compound metabolic process, small molecule metabolic process, ion binding and hydrolase activity are also the enriched GO terms based on shell-cloud genes of *L. crispatus.*

The above descriptions depict that while some of the biological functions in *L. crispatus* are totally conserved and some are strain-specific, many other functions as explained below are partially conserved and partially strain-specific. Similarly, for *L. iners*, functions related to topoisomerase, helicase, pyrimidine biosynthesis and bifunctional protein PyrR, cell cycle and penicillin-binding proteins, transpeptidase, GTP binding and homologous recombination are strain specific. While DNA binding, hydrolase, transferase, DNA damage and repair are partially core and partially strain-specific. The shell-cloud genes showed enrichment of functions such as DNA replication, translation and protein export, catalytic activity, pyrophosphate and ATPase activity, and nucleic acid-based metabolic process in *L. gasseri*, and nucleic acid metabolic process and purine nucleotide binding in *L. jensenii.* As such, we find that some of the core and soft-core based gene functions such as hydrolase, heterocyclic and aromatic compound metabolic process, and nitrogen compound metabolic process are the additional enriched GO terms in *L. gasseri* and *L. jensenii* based on the shell-cloud genes.

As mentioned in the preceding section, we found gene cluster gain/loss in *L. gasseri* and *L. jensenii* (Additional file 1: Table S1 sheet 9 and 10). Here we have also checked the functional enrichment related to those major gain/loss of gene clusters and found that the region-specific cluster in *L. gasseri* has a gain of functions such as amino acid and ABC transporter protein like ArtM/GltK/GlnP/TcyL/YhdX, S1 domain and ribonuclease D, dead/deah box helicase domain, biosynthesis of siderophore group nonriobosmal. However, no functional enrichment was found with the cluster of genes lost in *L. gasseri.* Similarly, *L jensenii* has region-specific cluster associated gain of functions such as MafB19, cytidine, APOBEC/CMP and deoxycytidylate like deaminase, zinc, and ATP binding domain, ABC transporter and macrolide export, and hydrolase activity, and loss of functions such as binding process associated with purine ribonucleotide, and metabolic process associated with the nucleobase-containing compound, carbohydrate derivative, cellular and heterocyclic organic compound, and catalytic activity.

### Variation of key functional attributes among the four predominant *Lactobacillus* species of vaginal microenvironment based on pangenome profile

The key attributes of lactobacilli that favour their dominance for a balanced and healthy vaginal microenvironment include plasma membrane integrity, adherence, aggregation, synthesis of exopolysaccharide (EPS), biosurfactant, bacteriocins, hydrogen peroxide and organic acids [1]. Many of the functionalities are correlated and involve a network of biological processes and molecular functions. Table 2 and Additional file 3: Table S2 provides a detail of the GO terms that defines the biological processes and molecular functions, including the associated genes based on the genome being analysed, that relates to the key attributes. Furthermore, it also shows the variations of the key attributes-related GO terms among the four predominant *Lactobacillus* species based on pangenome profile.

**Table 2:** Diversity of key attributes among the four predominant *Lactobacillus* species of vaginal microenvironment

Key functions	GO terms involved	Characteristic pattern in *L. crispatus*	Characteristic pattern in *L. iners*	Characteristic pattern in *L. gasseri*	Characteristic pattern in *L. jensenii*
Plasma membrane integrity	FAB	+(s)	-	-	-
Adherence, aggregation, and exopolysaccharide synthesis	CDB PB PTTA TA GM CA AgBP AFABP GaBP	+(sc/cl) - - +(s) +(s/cl) - - +(cl) -	+(cl) - - - - +(c) +(cl) +(c) +(cl)	+(sc/cl) - - +(c) +(sc/s/cl) - +(sc/s) - +(sc/s)	+(s/cl) +(sc) +(c) +(c) +(s) +(sc) - - -
Biosurfactant synthesis	Hydrolase transferase GCMP GcMP SM CMBP	+(s) +(c) +(sc) +(sc) +(s/cl) +(c)	+(sc/s) +(c) - - - +(sc/s/cl)	+(s) - +(sc) +(sc) - +(c/sc/s/cl)	+(s) +(c) - - - +(c)
Bacteriocin synthesis	ABCT TTA *nis*	- - +(s/cl)	+(cl) - -	+(s/cl) +(c) -	- +(c) -
Hydrogen peroxide, organic acid biosynthesis, and D/L-lactate metabolic processes	*pox* *nox* OSMP *ldh*	+(s/cl) +(cl) +(c) +(c)	- +(cl) +(s/cl) +(c/sc)	- - +(c/sc/s/cl) +(c/sc)	+(cl) - +(c) +(c)

• FAB – Fatty acid biosynthesis; CDB – Carbohydrate derivative binding; PB – Protein binding; PTTA – passive transmembrane transporter activity; TA – Transporter activity; GM - Galactose metabolism; CA - Chaperone activity; AgBP- Aminoglycan biosynthetic process; AFABP - Aspartate family amino acid biosynthetic process; GaBP - Glycosaminoglycan biosynthetic process ABCT – ABC transporter; TTA – Transmembrane transporter activity; *nis*– *nisin synthase*; *pox* – *pyruvate oxidase*; *Nox* – *NADH oxidase*; OSMP – Organic substance metabolic process; GCMP – Glycosyl compound metabolic process; GcMP - Glycosylceramide metabolic process; SM - Sphingolipid metabolism; CMBP – Cellular macromolecule biosynthetic process; *ldh – lactte dehydrogenase*

‘+’ and ‘-’ denotes present and absent respectively. ‘c’, ‘sc’, ‘s’, and ‘cl’ denotes core, soft-core, shell, and cloud respectively

Plasma membrane integrity is mostly associated with the fatty acid biosynthesis (FAB) pathway [22], and the pangenome profile reveals that the FAB pathway is a shell gene-associated enriched term solely found in *L. crispatus.* The adherence, aggregation, and EPS synthesis are interrelated functions that involve several biological processes such as, (i) carbohydrate derivative binding (CDB) and protein binding (PB) linked with bacterial competitive adhesion with the host’s receptor (ii) passive transmembrane transporter activity (PTTA) and transporter activity linked with adhesins, (iii) galactose metabolism (GM) linked with bacterial biofilm formation and flocculation, (iv) chaperone activity (CA) linked with fimbriae assembly, (v) aminoglycan biosynthetic process (AgBP) resulting in the formation of poly-N-acetyllactosamine (PNAG), (vi) aspartate family amino acid biosynthetic process (AFABP) linked with microbial surface components recognising adhesive matrix molecules (MSCRAMM) and (vii) glycosaminoglycan biosynthetic process (GaBP) linked with mucin secretion. Among them, *L. jensenii* showed enrichment of PTTA, TA, CA, and PB in core and soft-core genes, while CDB and GM in shell and cloud genes. *L. iners* showed enrichment of CA and AFABP in core genes, while CDB, AgBP, and GaBP in cloud genes. *L. gasseri* showed enrichment of TA in core genes, while CDB, GM, AgBP, and GaBP were among soft-core, shell, and cloud genes. Nevertheless, *L. crispatus* showed enrichment of CDB in soft-core and cloud genes, while TA, GM, and AFABP were in shell and cloud genes (Table 2).

The microbial synthesis of biosurfactant is a complex and biological process and has been associated with hydrolase and transferase activities, glycosyl compound metabolic process (GCMP) linked with glycolipids- biosurfactant synthesis, glycosylceramide metabolic process (GcMP) and sphingolipid metabolism (SM) linked to glycosphingolipids-biosurfactant synthesis, and cellular macromolecule biosynthetic process (CMBP) linked to high-molecular weight glycoprotein complexes and lipoproteins [23, 24]. Interestingly, *L. crispatus* pangenome showed enrichment of all biosurfactant-associated GO terms mostly in core and soft-core genes and fewer (hydrolase activity and SM) in shell and cloud genes. *L. gasseri* showed enrichment of CMBP throughout the pangenome profile, while GCMP and GcMP in soft-core genes, and hydrolase activity in shell genes. *L. iners* and *L. jensenii* only showed enrichment of hydrolase activity, transferase activity, and CMBP mostly in core and soft-core genes in *L. iners* and core and shell genes in *L. jensenii* (Table 2).

The biological processes associated with bacteriocin synthesis include ABC transporter (ABCT) and transmembrane transporter activity (TTA) and the gene *nisB* and *nisC* associated with the *nisin* biosynthesis protein [25, 26]. While TTA is an enriched term based on core genes of *L. jensenii* and *L. gasseri, L. crispatus* showed enrichment of *nisB* and *nisC* genes in shell and cloud cluster, and *L. iners.* showed enrichment of ABCT in cloud genes.

Hydrogen peroxide (H_2_O_2_), D/L-Lactate metabolic processes and organic acid synthesis induce antagonism against the pathogenic microbes, and the biological process associated with these functions includes the organic substance metabolic process (OSMP) and the gene *lactate dehydrogenase (ldh)* associated with lactic acid synthesis. Similarly, two other genes, viz. *pox* and *nox* associated with *pyruvate oxidase* and *NADH oxidase* are linked to hydrogen peroxide synthesis [27, 28]. OSMP is an enriched GO term mostly present in core genes of *L. crispatus*, *L. gasseri*, and *L. jensenii*, while *ldh* is present in core and soft-core gene cluster in all the lactobacilli. *pox* is present among the shell and cloud gene clusters of *L. crispatus* and *L. jensenii*, while *nox* is present in the cloud genes of *L. crispatus* and *L. iners.* Moreover, *phoU* gene related to phosphate acquisition [21] is found among the shell gene cluster of *L. crispatus*, and the core/soft-core gene cluster in *L. gasseri* and *L. jensenii*, but absent in *L. iners*.

## Discussion

The human microbiome project focuses on understanding the microbiota in several body parts such as oral, skin, vagina, gastrointestinal, and respiratory sites, involved in human health and diseases [29]. A unique attribute associated with a healthy vaginal microenvironment is less diversity of microbiota mostly dominated by lactobacilli [2]. A consensus of the previous studies showed that *L. crispatus*, *L. iners*, *L. gasseri*, and *L. jensenii* are dominant vaginal *Lactobacillus* species in healthy adult women [2–4]. The prevalence and sustenance of characteristic *Lactobacillus* species in healthy vaginal microenvironments, thereby acting as vaginal gatekeepers, need a comprehensive understanding. In particular, it is important to understand the genomic profile of the strains representing the four *Lactobacillus* species from different regions of the world and the key functionalities regulated by their genome. [1, 30].

Among the four predominant vaginal *Lactobacillus* species, the genome size is largest in *L. crispatus* (2.03 Mb) [31, 32] and smallest in *L. iners* (1.36 Mb) [33], while *L. gasseri* (1.87 Mb) and *L. jensenii* (1.59 Mb) have intermediate genome sizes. The shorter genome size has been previously linked with reduced metabolic capabilities due to a limited number of proteins encoded by the core and accessory genes of *L. iners* [18]. However, no previous studies provided a cumulative genome-based comparison among the four predominant *Lactobacillus* species. Our pangenome analysis provided a substantial understanding of the causes of the difference in lactobacilli genome size and reveals that the number of the core and soft-core genes marginally varies among the four species than the shell and cloud genes. However, considering the total number of genes, *L. iners* has more retention of core and soft-core genes, and *L. crispatus* has more retention of cloud genes. This provided a better understanding of population-exerted evolution in *L. crispatus* in humans, aside from niche-exerted evolution as defined previously [14].

Our study further revealed that alike *L. crispatus, L. jensenii* and *L. gasseri* strain evolution has also a significant link with varied human populations globally as revealed by a high percentage of their shell genes. Nevertheless, *L. iners* has maintained an equilibrium among the core and the cloud genes, which signifies its stable evolutionary process in the vaginal niche across a diverse population [33]. The ML phylogeny based on the core genes of all four species reveals that the although the clustering is population specific in some cases, there is also distinct cladding among the regional strains of a species, and few strains from diverse populations clustered cohesively. This intraspecies evolutionary pattern of the core genes is perhaps exerted by the stochastic factors in the vaginal microenvironment such as endogenous hormone level, sexual activity, hygiene practices, contraceptive and menstrual products, stress, and regional ethnicity [6, 34, 35].

Gene ontology enrichment analysis based on the core, soft-core, shell, and cloud genes revealed enrichment of either the whole biological process as ‘parent’ GO terms, or part of a biological process as ‘child’ GO term. We found that the basic cellular functions are enriched GO terms based on the core and soft-core genes of each of the species. While, considering the enrichment of unique key GO terms in a particular species, mention may be made of the enrichment of ion binding functions in soft-core, shell, and cloud genes of *L. crispatus*, which is likely related to iron chelating and transport system that helps in iron sequestering during menstruation [36]; a checkpoint to retard vaginal pathogens [18]. This particular feature is also evident as a major gain of function (biosynthesis of siderophore) in a cluster of strains from South Korea and the USA and therefore was found among the shell genes of *L. gasseri*.

Due to the high level of endogenous oestrogen, glycogen deposits maximally in the vaginal microenvironment, which the lactobacilli metabolize to lactic acid to induce a protective low pH condition [37]. Interestingly, we found that positive regulation of glycogen is an enriched function based on soft-core genes of *L. iners* and *L. jensenii*, and this supports the view that *L. crispatus* either lacks enzymes to directly degrade glycogen [38] or this trait may be strain-specific in *L. crispatus* [20] and *L. gasseri*. In another case, we found that an enriched function, divalent cation transport activity such as Mg^2+^ is involved in maintaining intracellular pH homeostasis in low pH vaginal microenvironment [39], and protection against oxidative stress [14, 40] is a core/soft-core based gene function in *L. crispatus L. iners*, and *L. gasseri*, while strain-specific in *L. jensenii.* Nevertheless, the enrichment of kinase/phosphorylase activity based on core/soft-core genes in *L. crispatrus, L. iners*, and *L. gasseri* and in shell genes of *L. jensenii* tentatively relates to a two-component system of bacteria involved in adapting to changing environmental conditions like oxidative stress due to hydrogen peroxide synthesis and cell envelope stress response [41, 42] that is common during antagonistic activity in the vaginal environment.

**Fig. 6:**
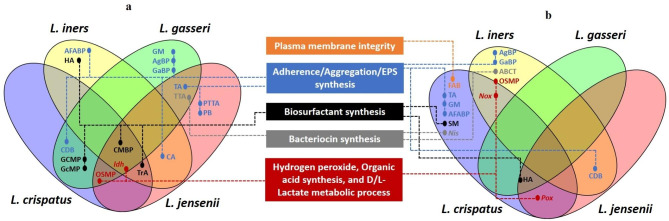
Schematic representation of the cellular processes associated with the key functional attributes and their distribution among the (**a**) core/soft-core genes, and (**b**) shell/cloud genes of the four predominant *Lactobacillus* species

The key attributes of the vaginal *Lactobacillus* species that makes them a successful protecting habitant in the vaginal environment against the pathogen have been summarised in a previous study [1]; it includes plasma membrane integrity, adherence, aggregation, EPS, biosurfactant, bacteriocin, hydrogen peroxide and organic acid synthesis. A summarised representation of the cellular processes associated with the key functional attributes and their distribution among the pangenome profile of the four predominant *Lactobacillus* species is shown schematically in Figs. 6aand b. Fatty acid biosynthesis (FAB), which is related to plasma membrane integrity and higher adaptability of a bacterial species to a dynamic or stressed environment [43], is an enriched GO term based on shell genes only in *L. crispatus*. Adherence, aggregation, and EPS synthesis are interrelated attributes and at least nine major cellular processes are associated with these attributes. Clearly, irrespective of the regional population, *L. gasseri* and *L. jensenii* carry these attributes as core functions, while in *L. crispatus* and *L. iners* these cellular functions are more strain specific. The biological process related to biosurfactant synthesis is a more common and mostly core function in *L. crispatus* with a single exception; sphingolipid metabolism (SM), which is strain specific. Nevertheless, a parent GO term “cellular macromolecule biosynthetic process” associated with biosurfactant synthesis is a common core function present in all four *Lactobacillus* species. Furthermore, bacteriocin synthesis is a core function in *L. gasseri* and *L. jensenii* and strain-specific in *L. crispatus* and *L. iners*. Nevertheless, hydrogen peroxide synthesis is strain-specific in *L. crispatus, L. iners*, and *L. jensenii*, while organic acid synthesis and D/L lactate metabolic process is a core function in *L. crispatus*, *L. jensenii*, *L. gasseri*, and *L. iners*. While a previous study [21] showed that gene (*phoU*) related to phosphate acquisition is uniquely present in *L. iners* strains of the vagina, our study showed that *phoU* gene is only present in *L. crispatus, L. gasseri*, and *L. jensenii*), while absent in *L. iners*. To cross compare this finding, we have checked the gene annotation file (GFF) corresponding to the genome assembly of *L. iners* (GCF_000149065, GCF_00149085, GCF_00149105, GCF_00149125, GCF_00149145, GCF_00185405, GCF_00191685, GCF_00204435), which further confirms the finding. The presence of *phoU* gene in *L. crispatus, L. gasseri*, and *L. jensenii* and its absence in *L. iners* may be associated with the physiology of the species in the vagina, however, it needs functional validation from future studies.

Overall, these findings reflect that neither a single species of *Lactobacillus* nor a strain bear all the favourable attributes altogether in its genome. This probably explains the reason for their co-habiting attribute of partitioning and sharing the vaginal niche, as found previously [18], to bring out combined protective functionality to the host. This further reflects the need to consider region-specific candidate strains of *Lactobacillus* to formulate an effective population-specific vaginal probiotic as a prophylactic measure against vaginal dysbiosis for women’s health.

## Materials and methods

### Data acquisition

For the exploration of various key features of predominant lactobacilli from different populations across the globe, we curated datasets for four major abundant *Lactobacillus* species, namely *L. crispatus*, *L. iners, L. gasseri* and *L. jensenii* from the NCBI database (https://www.ncbi.nlm.nih.gov/genome/browse). We examined all the datasets and considered vaginal and urine sources from human samples for further processing of the data. The information on the assembly of the genomes of *L. crispatus* (82), *L. iners* (25), *L. gasseri* (21), and *L. jensenii* (32) either as complete genome or as scaffolds or/contigs are provided in Additional file 1: Table 1 sheet 1–4. The nomenclature of the strains was based on the updated taxonomy in NCBI. The pangenome analysis covered publicly available lactobacilli genome datasets from the USA, the Netherlands, Italy, Belgium, China, India, Brazil, France, Ireland, Russia, South Korea, Japan, Thailand, and Sweden.

### Preparation of pangenome for different *Lactobacillus* species and phylogenetic reconstruction of the strains of each of the *Lactobacillus* species

The genomes were scanned to annotate the genes using a Prokka (version1.14.5) pipeline [44]. Briefly, ARAGORN [45], Barrnap [46] and Prodigal [46] annotated the genes related to tRNA, rRNA and protein-coding sequences. For annotating the protein coding genes, the minimum coverage on query protein was set at 80% with a similarity e-value cut-off of 1e-09. The protein-coding genes were further classified based on a domain-based search through HMMER and pfam database [47, 48]. For improved gene prediction, the minimum contig length was set to 200 bp along with a similarity cut-off of 1e-09. The annotated assemblies of each strain were used for the construction of large-scale pangenome to calculate core and accessory genes through Roary’s [49] pipeline. For annotating the core and accessory genes shared by the *Lactobacillus* strains, the Blastp percentage identity was set to 99% and 95%, respectively as defined previously [50]. The genomes were aligned through the progressive alignment method in MEGA 11 [51]. The Maximum Likelihood (ML) method and Tamura-Nei model [52] were used for the construction of phylogenetic trees, followed by tree refinement using Randomized Accelerated Maximum Likelihood (RAxML) [53]. Phandango [54] was used for proper visualization of the pangenome. This strategy allowed us to deduce the phylogenetic classification of the strains of each of the species and their corresponding pangenome.

### Functional enrichment analysis on the pangenome and assessment of gain-loss of the key genes linked with adaptability in the vaginal microenvironment

To understand the functional perspective of the genes, we used combinatorial results from ClueGo [55], EnrichR [56], and ShinyGO [57]. For ClueGo analysis, we selected *Lactobacillus* sp. as a reference organism for the annotation of the genes. The gene IDs were mapped automatically as per the existing bacterial database available in ClueGO. The visual style was selected based on node size significance. We selected biological processes and molecular functions for gene enrichment analysis against the updated gene ontology database. For robustness of the functional enrichments of the core and accessory genes, we used gene set libraries of enrichR and ShinyGO including GO biological process libraries 2021, and molecular function libraries 2021. The statistically significant GO terms (p-value ≤ 0.05) were further considered for downstream analysis. Cytoscape [58], an open-access bioinformatics software was used for visualization of gene cluster networks.

In this study, we primarily emphasized on the functional exploration of genes associated with adaptability in the vaginal microenvironment. Therefore, we curated the enriched GO terms associated with key functions like plasma membrane integrity, adherence, aggregation, exopolysaccharide, biosurfactant synthesis, synthesis of bacteriocins, hydrogen peroxide, and organic acids [1]. For this, we used the key functions and their associated terms from the literature as the ‘search term’ in the bacterial gene ontology browser [57]. We critically curated the GO term detail including the ‘parent’, ‘synonymous’, and nested terms (child GO term) under each of the GO terms to relate a particular GO term that represents the cascade of biological processes or molecular function associated with the key functionalities. Precisely, we have curated only those key attributes-related GO terms that are enriched based on the genomes analysed in this study. A supplementary of key attributes-related parent and child GO terms, associated genes, and the relevant literature that relates the GO term with the key functionalities is shown in Additional file 3: Table S2.

### Electronic supplementary material

Below is the link to the electronic supplementary material.

Supplementary Material 1

Supplementary Material 2

Supplementary Material 3

## Data Availability

The whole genome sequences of the lactobacilli analyzed in this study are downloaded from NCBI database and the details of their accession numbers are given in Additional file 1: Table S1.

## References

[1] Das S, Bhattacharjee MJ, Mukherjee AK, Khan MR. Recent advances in understanding of multifaceted changes in the vaginal microenvironment: implications in vaginal health and therapeutics. Crit Rev Microbiol 2022:1–27. 10.1080/1040841X.2022.2049696 35312419

[2] Ravel J, Gajer P, Abdo Z, Schneider GM, Koenig SS, McCulle SL, Karlebach S, Gorle R, Russell J, Tacket CO (2011). Vaginal microbiome of reproductive-age women. Proc Natl Acad Sci U S A.

[3] Chaban B, Links MG, Jayaprakash TP, Wagner EC, Bourque DK, Lohn Z, Albert AYK, van Schalkwyk J, Reid G, Hemmingsen SM (2014). Characterization of the vaginal microbiota of healthy canadian women through the menstrual cycle. Microbiome.

[4] Chen X, Lu Y, Chen T, Li R (2021). The female vaginal microbiome in Health and bacterial vaginosis. Front Cell Infect Microbiol.

[5] Amabebe E, Anumba DOC (2018). The Vaginal Microenvironment: the physiologic role of Lactobacilli. Front Med (Lausanne).

[6] Das Purkayastha S, Bhattacharya MK, Prasad HK, Upadhyaya H, Lala SD, Pal K, Das M, Sharma GD, Bhattacharjee MJ (2019). Contrasting diversity of vaginal lactobacilli among the females of Northeast India. BMC Microbiol.

[7] Sanders ME, Benson A, Lebeer S, Merenstein DJ, Klaenhammer TR (2018). Shared mechanisms among probiotic taxa: implications for general probiotic claims. Curr Opin Biotechnol.

[8] Roy H, Dare K, Ibba M (2009). Adaptation of the bacterial membrane to changing environments using aminoacylated phospholipids. Mol Microbiol.

[9] Boris S, Suarez JE, Vazquez F, Barbes C (1998). Adherence of human vaginal lactobacilli to vaginal epithelial cells and interaction with uropathogens. Infect Immun.

[10] Kmet V, Lucchini F (1997). Aggregation-promoting factor in human vaginal Lactobacillus strains. FEMS Immunol Med Microbiol.

[11] Phukan N, Brooks AES, Simoes-Barbosa A. A cell surface aggregation-promoting factor from Lactobacillus gasseri contributes to inhibition of Trichomonas vaginalis Adhesion to Human Vaginal Ectocervical cells. Infect Immun 2018, 86(8). 10.1128/IAI.00907-17 PMC6056882 29784856

[12] Mokoena MP. Lactic acid Bacteria and their bacteriocins: classification, biosynthesis and applications against Uropathogens: a Mini-Review. Molecules 2017, 22(8). 10.3390/molecules22081255 PMC6152299 28933759

[13] Larsen B, Monif GR (2001). Understanding the bacterial flora of the female genital tract. Clin Infect Dis.

[14] Zhang Q, Zhang L, Ross P, Zhao J, Zhang H, Chen W. Comparative Genomics of Lactobacillus crispatus from the gut and Vagina reveals genetic diversity and lifestyle adaptation. Genes (Basel) 2020, 11(4). 10.3390/genes11040360 PMC7230607 32230824

[15] Allonsius CN, van den Broek MFL, De Boeck I, Kiekens S, Oerlemans EFM, Kiekens F, Foubert K, Vandenheuvel D, Cos P, Delputte P (2017). Interplay between Lactobacillus rhamnosus GG and Candida and the involvement of exopolysaccharides. Microb Biotechnol.

[16] Morais IMC, Cordeiro AL, Teixeira GS, Domingues VS, Nardi RMD, Monteiro AS, Alves RJ, Siqueira EP, Santos VL (2017). Biological and physicochemical properties of biosurfactants produced by Lactobacillus jensenii P6A and Lactobacillus gasseri P65. Microb Cell Fact.

[17] Mancabelli L, Mancino W, Lugli GA, Milani C, Viappiani A, Anzalone R, Longhi G, van Sinderen D, Ventura M, Turroni F. Comparative genome analyses of Lactobacillus crispatus isolated from different ecological niches reveal an environmental adaptation of this species to the human vaginal environment. Appl Environ Microbiol 2021. 10.1128/AEM.02899-20 PMC8091109 33579685

[18] France MT, Mendes-Soares H, Forney LJ (2016). Genomic comparisons of Lactobacillus crispatus and Lactobacillus iners reveal potential ecological drivers of Community Composition in the Vagina. Appl Environ Microbiol.

[19] Fontana F, Alessandri G, Lugli GA, Mancabelli L, Longhi G, Anzalone R, Viappiani A, Ventura M, Turroni F, Milani C. Probiogenomics Analysis of 97 Lactobacillus crispatus strains as a Tool for the identification of Promising Next-Generation Probiotics. Microorganisms 2020, 9(1). 10.3390/microorganisms9010073 PMC7824148 33396617

[20] van der Veer C, Hertzberger RY, Bruisten SM, Tytgat HLP, Swanenburg J, de Angelino-Bart K, Schuren A, Molenaar F, Reid D, de Vries G (2019). Comparative genomics of human Lactobacillus crispatus isolates reveals genes for glycosylation and glycogen degradation: implications for in vivo dominance of the vaginal microbiota. Microbiome.

[21] Mendes-Soares H, Suzuki H, Hickey RJ, Forney LJ (2014). Comparative functional genomics of Lactobacillus spp. reveals possible mechanisms for specialization of vaginal lactobacilli to their environment. J Bacteriol.

[22] Zhang YM, Rock CO (2008). Membrane lipid homeostasis in bacteria. Nat Rev Microbiol.

[23] Shu Q, Lou H, Wei T, Liu X, Chen Q. Contributions of glycolipid biosurfactants and glycolipid-modified materials to Antimicrobial Strategy: a review. Pharmaceutics 2021, 13(2). 10.3390/pharmaceutics13020227 PMC7914807 33562052

[24] Eras-Munoz E, Farre A, Sanchez A, Font X, Gea T (2022). Microbial biosurfactants: a review of recent environmental applications. Bioengineered.

[25] Beis K, Rebuffat S (2019). Multifaceted ABC transporters associated to microcin and bacteriocin export. Res Microbiol.

[26] Kleerebezem M (2004). Quorum sensing control of lantibiotic production; nisin and subtilin autoregulate their own biosynthesis. Peptides.

[27] Cornacchione LP, Hu LT (2020). Hydrogen peroxide-producing pyruvate oxidase from Lactobacillus delbrueckii is catalytically activated by phosphotidylethanolamine. BMC Microbiol.

[28] Groeger G, Mackey AM, Pettigrew CA, Bhatt L, Cotter TG (2009). Stress-induced activation of Nox contributes to cell survival signalling via production of hydrogen peroxide. J Neurochem.

[29] Integrative HMPRNC (2019). The Integrative Human Microbiome Project. Nature.

[30] Ma B, Forney LJ, Ravel J (2012). Vaginal microbiome: rethinking health and disease. Annu Rev Microbiol.

[31] Price TK, Shaheen M, Kalesinskas L, Malki K, Hilt EE, Putonti C, Wolfe AJ. Draft genome sequence of a urinary isolate of Lactobacillus crispatus. Genome Announc 2016, 4(6). 10.1128/genomeA.01278-16 PMC5137400 27908986

[32] Khan F, Miller-Ensminger T, Voukadinova A, Wolfe AJ, Putonti C. Draft genome sequence of Lactobacillus crispatus UMB1163, isolated from the female urinary tract. Microbiol Resour Announc 2020, 9(23). 10.1128/MRA.00404-20 PMC7272555 32499346

[33] Kwak W, Han YH, Seol D, Kim H, Ahn H, Jeong M, Kang J, Kim H, Kim TH (2020). Complete genome of Lactobacillus iners KY using Flongle provides insight into the genetic background of optimal adaption to Vaginal Econiche. Front Microbiol.

[34] Hickey RJ, Zhou X, Pierson JD, Ravel J, Forney LJ (2012). Understanding vaginal microbiome complexity from an ecological perspective. Translational Research: The Journal of Laboratory and Clinical Medicine.

[35] Vargas-Robles D, Morales N, Rodríguez I, Nieves T, Godoy-Vitorino F, Alcaraz LD, Pérez ME, Ravel J, Forney LJ (2020). Domínguez-Bello MG: changes in the vaginal microbiota across a gradient of urbanization. Sci Rep.

[36] Hallberg L, Högdahl AM, Nilsson L, Rybo G (1966). Menstrual blood loss and iron deficiency. Acta Med Scand.

[37] Mirmonsef P, Hotton AL, Gilbert D, Burgad D, Landay A, Weber KM, Cohen M, Ravel J, Spear GT (2014). Free glycogen in vaginal fluids is associated with Lactobacillus colonization and low vaginal pH. PLoS ONE.

[38] Ojala T, Kankainen M, Castro J, Cerca N, Edelman S, Westerlund-Wikstrom B, Paulin L, Holm L, Auvinen P (2014). Comparative genomics of Lactobacillus crispatus suggests novel mechanisms for the competitive exclusion of Gardnerella vaginalis. BMC Genomics.

[39] Shabayek S, Spellerberg B (2017). Acid stress response mechanisms of Group B Streptococci. Front Cell Infect Microbiol.

[40] Cotter PD, Hill C (2003). Surviving the acid test: responses of gram-positive bacteria to low pH. Microbiol Mol Biol Rev.

[41] Zhao Z, Peng T, Oh JI, Glaeser J, Weber L, Li Q, Klug G (2019). A response regulator of the OmpR family is part of the regulatory network controlling the oxidative stress response of Rhodobacter sphaeroides. Environ Microbiol Rep.

[42] Stock AM, Robinson VL, Goudreau PN (2000). Two-component signal transduction. Annu Rev Biochem.

[43] Kengmo Tchoupa A, Eijkelkamp BA, Peschel A (2022). Bacterial adaptation strategies to host-derived fatty acids. Trends Microbiol.

[44] Seemann T (2014). Prokka: rapid prokaryotic genome annotation. Bioinformatics.

[45] Laslett D, Canback B (2004). ARAGORN, a program to detect tRNA genes and tmRNA genes in nucleotide sequences. Nucleic Acids Res.

[46] Hyatt D, Chen GL, Locascio PF, Land ML, Larimer FW, Hauser LJ (2010). Prodigal: prokaryotic gene recognition and translation initiation site identification. BMC Bioinformatics.

[47] Potter SC, Luciani A, Eddy SR, Park Y, Lopez R, Finn RD (2018). HMMER web server: 2018 update. Nucleic Acids Res.

[48] Mistry J, Chuguransky S, Williams L, Qureshi M, Salazar GA, Sonnhammer ELL, Tosatto SCE, Paladin L, Raj S, Richardson LJ (2021). Pfam: the protein families database in 2021. Nucleic Acids Res.

[49] Page AJ, Cummins CA, Hunt M, Wong VK, Reuter S, Holden MT, Fookes M, Falush D, Keane JA, Parkhill J (2015). Roary: rapid large-scale prokaryote pan genome analysis. Bioinformatics.

[50] Sitto F, Battistuzzi FU (2020). Estimating pangenomes with Roary. Mol Biol Evol.

[51] Tamura K, Stecher G, Kumar S (2021). MEGA11: Molecular Evolutionary Genetics Analysis Version 11. Mol Biol Evol.

[52] Tamura K, Nei M (1993). Estimation of the number of nucleotide substitutions in the control region of mitochondrial DNA in humans and chimpanzees. Mol Biol Evol.

[53] Stamatakis A (2014). RAxML version 8: a tool for phylogenetic analysis and post-analysis of large phylogenies. Bioinformatics.

[54] Hadfield J, Croucher NJ, Goater RJ, Abudahab K, Aanensen DM, Harris SR (2018). Phandango: an interactive viewer for bacterial population genomics. Bioinformatics.

[55] Bindea G, Mlecnik B, Hackl H, Charoentong P, Tosolini M, Kirilovsky A, Fridman WH, Pages F, Trajanoski Z, Galon J (2009). ClueGO: a Cytoscape plug-in to decipher functionally grouped gene ontology and pathway annotation networks. Bioinformatics.

[56] Kuleshov MV, Jones MR, Rouillard AD, Fernandez NF, Duan Q, Wang Z, Koplev S, Jenkins SL, Jagodnik KM, Lachmann A (2016). Enrichr: a comprehensive gene set enrichment analysis web server 2016 update. Nucleic Acids Res.

[57] Ge SX, Jung D, Yao R (2020). ShinyGO: a graphical gene-set enrichment tool for animals and plants. Bioinformatics.

[58] Shannon P, Markiel A, Ozier O, Baliga NS, Wang JT, Ramage D, Amin N, Schwikowski B, Ideker T (2003). Cytoscape: a software environment for integrated models of biomolecular interaction networks. Genome Res.

