# Supplementary material for: Comparative pangenomic analysis of predominant human vaginal lactobacilli strains towards population-specific adaptation: understanding the role in sustaining a balanced and healthy vaginal microenvironment
Source: BMC Genomics. 2023 Sep 22;24:565. doi: 10.1186/s12864-023-09665-y (PMC10517566; doi:10.1186/s12864-023-09665-y)
Supplement: Supplementary file 2 — Supplementary Material 2 [file 12864_2023_9665_MOESM2_ESM.pdf]

Lactobacillus crispatus

Significant GO terms (p<=0.05) of *L. crispatus* genes	Core	Soft-core	Shell	Cloud
Aspartate family amino acid biosynthetic process	■			■
ATP-binding	■		■	■
Cytoplasm	■	■	■	■
DNA damage			■	■
DNA repair			■	■
DNA-binding			■	■
Endopeptidase activity		■		■
Galactose metabolism, and Domain of unknown function			■	■
Galactose metabolism, and Sucrose phosphorylase			■	■
Ligase	■			■
macromolecule biosynthetic process	■	■		
Magnesium	■			■
Metal-binding	■	■	■	■
mixed, incl. Ribonucleoprotein, and Cytoplasm	■			■
Nucleotide-binding	■		■	■
Protein biosynthesis	■		■	
Sphingolipid metabolism, and Beta-galactosidase			■	■
Transferase			■	■
Zinc			■	■
amide biosynthetic process	■			
carboxylic acid metabolic process	■			
Cell cycle	■			
Cell division	■			
Cell division, and D-Alanine metabolism	■			
cellular amide metabolic process	■			
cellular amino acid metabolic process	■			
cellular biosynthetic process	■			
cellular component biogenesis	■			
cellular component disassembly	■			
cellular macromolecule biosynthetic process	■			
cellular macromolecule metabolic process	■			
cellular nitrogen compound biosynthetic process	■			
cellular nitrogen compound metabolic process	■			
cellular protein metabolic process	■			
gene expression	■			
intracellular non-membrane-bounded organelle	■			
intracellular organelle	■			
large ribosomal subunit	■			
macromolecule metabolic process	■			
mixed, incl. Cell division, and D-Alanine metabolism	■			
mixed, incl. Ribonucleoprotein, and Protein biosynthesis	■			
ncRNA metabolic process	■			
ncRNA processing	■			

Significant GO terms (p<=0.05) of *L. crispatus* genes	Core	Soft-core	Shell	Cloud
non-membrane-bounded organelle	■			
nucleic acid binding	■			
nucleobase-containing compound kinase activity	■			
nucleoside monophosphate kinase activity	■			
nucleoside monophosphate phosphorylation	■			
organic acid metabolic process	■			
organic substance biosynthetic process	■			
organonitrogen compound biosynthetic process	■			
organonitrogen compound metabolic process	■			
oxoacid metabolic process	■			
pentose metabolic process	■			
pentose-phosphate shunt	■			
pentose-phosphate shunt, non-oxidative branch	■			
peptide biosynthetic process	■			
peptide metabolic process	■			
phosphotransferase activity, phosphate group as acceptor	■			
protein metabolic process	■			
protein-containing complex disassembly	■			
protein-containing complex subunit organization	■			
purine-containing compound salvage	■			
Ribonucleoprotein	■			
ribonucleoprotein complex biogenesis	■			
Ribonucleoprotein, and Protein biosynthesis	■			
Ribosomal protein	■			
ribosomal small subunit biogenesis	■			
ribosomal subunit	■			
ribosome	■			
ribosome biogenesis	■			
Ribosome, and Bacterial secretion system	■			
Ribosome, and Elongation factor	■			
Ribosome, and Protein biosynthesis	■			
RNA binding	■			
RNA modification	■			
RNA processing	■			
rRNA binding	■			
small ribosomal subunit	■			
translation	■			
tRNA binding	■			
tRNA metabolic process	■			
tRNA modification	■			
tRNA processing	■			
glycoside catabolic process		■		
glycoside metabolic process		■		
glycosyl compound catabolic process		■		

Significant GO terms (p<=0.05) of *L. crispatus* genes	Core	Soft-core	Shell	Cloud
glycosyl compound metabolic process		■		
glycosylceramide catabolic process		■		
glycosylceramide metabolic process		■		
intrinsic component of plasma membrane		■		
negative regulation of monooxygenase activity		■		
nucleoside metabolic process		■		
pyridine-containing compound metabolic process		■		
pyrimidine deoxyribonucleotide biosynthetic process		■		
regulation of inositol phosphate biosynthetic process		■		
tRNA synthetases class II (D, K and N)		■		
fatty acid biosynthetic process			■	
fatty acid metabolic process			■	
monocarboxylic acid biosynthetic process			■	
nucleoside bisphosphate biosynthetic process			■	
purine nucleoside bisphosphate biosynthetic process			■	
ribonucleoside bisphosphate biosynthetic process			■	
PTS EIIA type-2 domain			■	
Phosphotransferase/anion transporter			■	
Phosphoenolpyruvate-dependent sugar phosphotransferase system, EIIA 2			■	
Hydrolase			■	
mixed, incl. Lipid biosynthesis, and Glycerophosphoryl diester phosphodiesterase			■	
galactose catabolic process			■	
galactose metabolic process			■	
Amino-acid biosynthesis				■
aspartate family amino acid metabolic process				■
carbon-nitrogen ligase activity, with glutamine as amido-N-donor				■
Cell membrane				■
DEAD/DEAH box helicase				■
DEAD-like helicases superfamily				■
DNA repair, and DNA helicase DnaB, N-terminal/DNA primase DnaG, C-terminal				■
DNA replication				■
glucuronate catabolic process				■
glucuronate catabolic process to xylulose 5-phosphate				■
Helicase conserved C-terminal domain				■
Helicase superfamily 1/2, ATP-binding domain				■
Helicase, C-terminal				■
Helicase/UvrB, N-terminal				■
Homologous recombination, and Excision nuclease				■
hydroxymethyl-, formyl- and related transferase activity				■
IMP biosynthetic process				■
IMP metabolic process				■

Significant GO terms (p<=0.05) of *L. crispatus* genes	Core	Soft-core	Shell	Cloud
intramolecular transferase activity				■
ligase activity, forming carbon-nitrogen bonds				■
mixed, incl. Homologous recombination, and DNA topoisomerase, type IIA-like domain superfamily				■
monocarboxylic acid metabolic process				■
nucleoside monophosphate biosynthetic process				■
nucleoside monophosphate metabolic process				■
phosphoribosylformylglycinamidine synthase activity				■
phosphorylation				■
phosphotransferase activity, alcohol group as acceptor				■
phosphotransferase activity, for other substituted phosphate groups				■
P-loop containing nucleoside triphosphate hydrolase				■
purine nucleoside monophosphate biosynthetic process				■
purine nucleoside monophosphate metabolic process				■
purine nucleotide biosynthetic process				■
purine nucleotide metabolic process				■
purine ribonucleoside monophosphate biosynthetic process				■
purine ribonucleoside monophosphate metabolic process				■
purine ribonucleotide biosynthetic process				■
purine ribonucleotide metabolic process				■
purine-containing compound biosynthetic process				■
purine-containing compound metabolic process				■
ribonucleoside monophosphate biosynthetic process				■
ribonucleoside monophosphate metabolic process				■
ribonucleotide biosynthetic process				■
ribonucleotide metabolic process				■
ribose phosphate biosynthetic process				■
ribose phosphate metabolic process				■
transferase complex				■
Type III restriction enzyme, res subunit				■
xylulose 5-phosphate biosynthetic process				■
Zinc-finger				■

Figure S1: Significant functional enrichment analysis of the GOs using ClueGo, EnrichR, and ShinyGO search platform using the core, soft-core, shell, and cloud genes of *L. crispatus*

Lactobacillus iners

Significant GO terms (p<=0.05) of L. iners genes	Core	Soft-core	Shell	Cloud
Aminoacyl-tRNA synthetase				
ATP-binding				
Cell cycle				
Cell division				
Cytoplasm				
Ligase				
Metal-binding				
mixed, incl. Ribonucleoprotein, and Protein biosynthesis				
Nucleotide-binding				
Protein biosynthesis				
ribonucleotide metabolic process				
ribose phosphate metabolic process				
Ribosome, and Protein biosynthesis				
RNA-binding				
rRNA-binding				
adenyl nucleotide binding				
adenyl ribonucleotide binding				
amide biosynthetic process				
amino acid activation				
Aminoacyl-tRNA biosynthesis				
aminoacyl-tRNA ligase activity				
anion binding				
ATP binding				
ATPase activity				
catalytic activity, acting on a tRNA				
cellular amide metabolic process				
cellular component organization				
cellular macromolecule biosynthetic process				
cellular macromolecule metabolic process				
cellular nitrogen compound biosynthetic process				
cellular protein metabolic process				
cellular response to DNA damage stimulus				
cellular response to stress				
chromosome organization				
DNA conformation change				
DNA damage				
DNA metabolic process				
DNA repair				
exonuclease activity				
gene expression				
Homologous recombination				
Hydrolase				
hydrolase activity, acting on acid anhydrides				
hydrolase activity, acting on acid anhydrides, in phosphorus-containing anhydrides				
hydrolase activity, acting on ester bonds				

Significant GO terms (p<=0.05) of L. iners genes	Core	Soft-core	Shell	Cloud
ligase activity, forming carbon-oxygen bonds				
macromolecule biosynthetic process				
macromolecule metabolic process				
ncRNA metabolic process				
Nuclease				
nuclease activity				
nucleic acid metabolic process				
nucleic acid phosphodiester bond hydrolysis				
nucleoside phosphate binding				
nucleoside-triphosphatase activity				
nucleotide binding				
organelle organization				
organic substance catabolic process				
pentose metabolic process (GO:0019321)				
pentose-phosphate shunt, non-oxidative branch (GO:0009052)				
peptide biosynthetic process				
peptide metabolic process				
protein metabolic process				
purine nucleotide binding				
purine ribonucleoside triphosphate binding				
purine ribonucleotide binding				
pyrophosphatase activity				
ribonucleotide binding				
RNA metabolic process				
translation				
tRNA aminoacylation				
tRNA aminoacylation for protein translation				
tRNA metabolic process				
3'-5' exonuclease activity				
amide transport				
Bacterial secretion system				
carbohydrate derivative metabolic process				
cellular aromatic compound metabolic process				
cellular biosynthetic process				
cellular macromolecule localization				
cellular protein localization				
chromosome				
Coiled coil				
DNA replication				
DNA-binding				
DNA-dependent ATPase activity				
DNA-dependent DNA replication				
establishment of localization in cell				
establishment of protein localization				
establishment of protein localization to membrane				

Significant GO terms (p<=0.05) of L. iners genes	Core	Soft-core	Shell	Cloud
GTPase activity				
heterocycle metabolic process				
Homologous recombination, and DNA repair				
Homologous recombination, and DNA replication				
Homologous recombination, and PD-(D/E)XK nuclease superfamily				
Homologous recombination, and SOS response				
intracellular protein transport				
intracellular transport				
mixed, incl. Homologous recombination, and DNA repair				
nucleic acid binding				
nucleobase-containing compound metabolic process				
nucleoside binding				
organic cyclic compound metabolic process				
organic substance biosynthetic process				
peptide transport				
P-loop containing nucleoside triphosphate hydrolase				
Protein export				
protein localization				
protein localization to membrane				
protein targeting				
protein transport				
ribonucleoside binding				
aspartate family amino acid biosynthetic process				
aspartate family amino acid metabolic process				
carbon-nitrogen ligase activity, with glutamine as amido-N-donor				
endopeptidase activity				
hydroxymethyl-, formyl- and related transferase activity				
IMP biosynthetic process				
IMP metabolic process				
intramolecular transferase activity				
ligase activity, forming carbon-nitrogen bonds				
Magnesium				
mixed, incl. Ribonucleoprotein, and Cytoplasm				
monocarboxylic acid metabolic process				
nucleoside monophosphate biosynthetic process				
nucleoside monophosphate metabolic process				
Nucleotidyltransferase				
phosphoribosylformylglycinamidine synthase activity				
phosphorylation				
phosphotransferase activity, alcohol group as acceptor				
phosphotransferase activity, for other substituted phosphate groups				
purine nucleoside monophosphate biosynthetic process				

Significant GO terms (p<=0.05) of L. iners genes	Core	Soft-core	Shell	Cloud
purine nucleoside monophosphate metabolic process				
purine nucleotide biosynthetic process				
purine nucleotide metabolic process				
purine ribonucleoside monophosphate biosynthetic process				
purine ribonucleoside monophosphate metabolic process				
purine ribonucleotide biosynthetic process				
purine ribonucleotide metabolic process				
purine-containing compound biosynthetic process				
purine-containing compound metabolic process				
pyrimidine deoxyribonucleotide catabolic process (GO:0009223)				
Ribonucleoprotein				
Ribonucleoprotein, and Protein biosynthesis				
ribonucleoside monophosphate biosynthetic process				
ribonucleoside monophosphate metabolic process				
ribonucleotide biosynthetic process				
ribose phosphate biosynthetic process				
Ribosomal protein				
Ribosome				
Ribosome, and Bacterial secretion system				
Ribosome, and Elongation factor				
transferase complex				
tRNA-binding				
Beta-grasp domain superfamily				
bone resorption (GO:0045453)				
cAMP metabolic process (GO:0046058)				
cation binding				
cellular catabolic process				
Creatinase, N-terminal, and POT family				
DALR anticodon binding				
DALR anticodon binding domain				
intracellular non-membrane-bounded organelle				
intracellular organelle				
macromolecule biosynthetic process (GO:0009059)				
macromolecule catabolic process				
metal ion binding				
mixed, incl. Chaperone, and Repressor				
mixed, incl. Endonuclease, and PPIC-type PPIASE domain				
mixed, incl. TRCF domain, and MviN-like protein				
mixed, incl. Uncharacterized ACR, COG1399, and Protein with unknown function UPF0154				
mostly uncharacterized, incl. Endonuclease, and Foldase protein PrsA				
mostly uncharacterized, incl. Kinase/pyrophosphorylase, and Glycine-tRNA synthetase, heterodimeric				
mostly uncharacterized, incl. Nuclease, and Foldase protein PrsA				
mostly uncharacterized, incl. PPIC-type PPIASE domain, and HIT domain				
mostly uncharacterized, incl. YspA SLOG family, and Regulatory protein Spx				
ncRNA processing				
non-membrane-bounded organelle				
pentose catabolic process (GO:0019323)				
pentose-phosphate shunt (GO:0006098)				
positive regulation of alcohol biosynthetic process (GO:1902932)				
positive regulation of cellular carbohydrate metabolic process (GO:0010676)				
positive regulation of glycogen biosynthetic process (GO:0045725)				
positive regulation of glycogen metabolic process (GO:0070875)				
positive regulation of inositol phosphate biosynthetic process (GO:0060732)				

Significant GO terms (p<=0.05) of L. iners genes	Core	Soft-core	Shell	Cloud
regulation of inositol phosphate biosynthetic process (GO:0010919)				
regulation of synaptic vesicle priming (GO:0010807)				
Ribonuclease III domain				
Ribonuclease III family				
Ribonuclease III, endonuclease domain superfamily				
rRNA binding				
TGS-like				
transition metal ion binding				
tRNA processing				
Zinc				
zinc ion binding				
Cell shape				
cellular response to lipoteichoic acid (GO:0071223)				
cellular response to nicotine (GO:0071316)				
DNA topoisomerase, type IIA				
DNA topoisomerase, type IIA, subunit B				
DNA topoisomerase, type IIA-like domain superfamily, and S4 domain				
Exonuclease				
GTP binding				
GTP-binding				
guanyl nucleotide binding				
guanyl ribonucleotide binding				
Helicase				
mixed, incl. Peptidoglycan biosynthesis, and Cell cycle protein				
pteridine-containing compound biosynthetic process (GO:0042559)				
purine nucleoside binding				
purine ribonucleoside binding				
Pyrimidine biosynthesis				
Pyrimidine biosynthesis, and Uridine kinase				
Quorum sensing				
regulation of miRNA metabolic process (GO:2000628)				
response to angiotensin (GO:1990776)				
response to lipoteichoic acid (GO:0070391)				
response to peptidoglycan (GO:0032494)				
Signal recognition particle, SRP54 subunit, GTPase domain				
adenylyltransferase activity				
aminoglycan biosynthetic process				
aminoglycan metabolic process				
carbohydrate derivative biosynthetic process				
catalytic activity, acting on a rRNA				
cell wall biogenesis				
cell wall macromolecule biosynthetic process				
cell wall macromolecule metabolic process				
cell wall organization				
cellular component biogenesis				
cellular component macromolecule biosynthetic process				
cellular response to external stimulus				
cellular response to extracellular stimulus				
DEAD-like helicases superfamily				
deoxyribonuclease activity				
D-Glutamine and D-glutamate metabolism				
DNA binding				
DNA biosynthetic process				
DNA duplex unwinding				
DNA geometric change				
DNA polymerase activity				
DNA topoisomerase activity				
DNA topoisomerase type II (double strand cut, ATP-hydrolyzing) activity				
DNA topological change				
DNA-directed DNA polymerase				
DNA-directed DNA polymerase activity				
double-stranded DNA binding				
endodeoxyribonuclease activity				
endodeoxyribonuclease complex				
endonuclease complex				
excinuclease ABC activity				
excinuclease repair complex				
external encapsulating structure organization				
glycosaminoglycan biosynthetic process				
glycosaminoglycan metabolic process				
tRNA binding				

Significant GO terms (p<=0.05) of L. iners genes	Core	Soft-core	Shell	Cloud
Helicase				
mixed, incl. Peptidoglycan biosynthesis, and Cell cycle protein				
pteridine-containing compound biosynthetic process (GO:0042559)				
purine nucleoside binding				
purine ribonucleoside binding				
Pyrimidine biosynthesis				
Pyrimidine biosynthesis, and Uridine kinase				
Quorum sensing				
regulation of miRNA metabolic process (GO:2000628)				
response to angiotensin (GO:1990776)				
response to lipoteichoic acid (GO:0070391)				
response to peptidoglycan (GO:0032494)				
Signal recognition particle, SRP54 subunit, GTPase domain				
adenylyltransferase activity				
aminoglycan biosynthetic process				
aminoglycan metabolic process				
carbohydrate derivative biosynthetic process				
catalytic activity, acting on a rRNA				
cell wall biogenesis				
cell wall macromolecule biosynthetic process				
cell wall macromolecule metabolic process				
cell wall organization				
cellular component biogenesis				
cellular component macromolecule biosynthetic process				
cellular response to external stimulus				
cellular response to extracellular stimulus				
DEAD-like helicases superfamily				
deoxyribonuclease activity				
D-Glutamine and D-glutamate metabolism				
DNA binding				
DNA biosynthetic process				
DNA duplex unwinding				
DNA geometric change				
DNA polymerase activity				
DNA topoisomerase activity				
DNA topoisomerase type II (double strand cut, ATP-hydrolyzing) activity				
DNA topological change				
DNA-directed DNA polymerase				
DNA-directed DNA polymerase activity				
double-stranded DNA binding				
endodeoxyribonuclease activity				
endodeoxyribonuclease complex				
endonuclease complex				
excinuclease ABC activity				
excinuclease repair complex				
external encapsulating structure organization				
glycosaminoglycan biosynthetic process				
glycosaminoglycan metabolic process				
helicase activity				
Helicase, C-terminal				
macromolecule methylation				
Mismatch repair				
mixed, incl. Ribosome, and Protein biosynthesis				
N-methyltransferase activity				
Nucleic acid-binding, OB-fold				
nucleobase-containing compound biosynthetic process				
nucleotide-excision repair				
nucleotidyltransferase activity				
organonitrogen compound biosynthetic process				
organonitrogen compound metabolic process				
peptidoglycan biosynthetic process				
peptidoglycan metabolic process				
peptidoglycan-based cell wall biogenesis				
regulation of developmental process				
response to extracellular stimulus				
RNA binding				
rRNA methyltransferase activity				
S-adenosylmethionine-dependent methyltransferase activity				
SOS response				
Topoisomerase				
transferase activity, transferring phosphorus-containing groups				
tRNA binding				

Figure S2: Significant functional enrichment analysis of the GOs using ClueGo, EnrichR, and ShinyGO search platform using the core, soft-core, shell, and cloud genes of *L. iners*

Lactobacillus gasseri

Significant GO terms (p<=0.05) of L. gaesseri genes	Core	Soft-core	Shell	Cloud
5'-3' RNA polymerase activity				
adenyl nucleotide binding				
adenyl ribonucleotide binding				
ADP metabolic process				
amide biosynthetic process				
amino acid activation				
Aminoacyl-tRNA biosynthesis				
aminoacyl-tRNA ligase activity				
Aminoacyl-tRNA synthetase				
anion binding				
ATP binding				
ATP generation from ADP				
ATP-binding				
carbohydrate catabolic process				
carbohydrate derivative biosynthetic process				
carbohydrate derivative metabolic process				
carboxylic ester hydrolase activity				
catalytic activity, acting on a tRNA				
cation binding				
cell wall biogenesis				
cell wall macromolecule biosynthetic process				
cell wall macromolecule metabolic process				
cell wall organization				
cellular amide metabolic process				
cellular aromatic compound metabolic process				
cellular biosynthetic process				
cellular component biogenesis				
cellular component macromolecule biosynthetic process				
cellular component organization				
cellular macromolecule biosynthetic process				
cellular macromolecule metabolic process				
cellular nitrogen compound biosynthetic process				
cellular nitrogen compound metabolic process				
cellular protein metabolic process				
cellular response to external stimulus				
cellular response to extracellular stimulus				
Chromosome				
Cytoplasm				
DNA conformation change				
DNA replication				
DNA-dependent DNA replication				
DNA-directed 5'-3' RNA polymerase activity				
endoribonuclease activity				
external encapsulating structure organization				
gene expression				
glycolytic process				
GTP binding				
GTPase activity				
guanyl nucleotide binding				
guanyl ribonucleotide binding				

Significant GO terms (p<=0.05) of L. gaesseri genes	Core	Soft-core	Shell	Cloud
heterocycle metabolic process				
intracellular non-membrane-bounded organelle				
intracellular organelle				
large ribosomal subunit				
Ligase				
ligase activity, forming carbon-oxygen bonds				
macromolecule biosynthetic process				
macromolecule metabolic process				
magnesium ion binding				
metal ion binding				
mixed, incl. Ribonucleoprotein, and Cytoplasm				
ncRNA metabolic process				
ncRNA processing				
non-membrane-bounded organelle				
nucleic acid binding				
nucleic acid metabolic process				
nucleobase-containing compound biosynthetic process				
nucleobase-containing compound metabolic process				
nucleobase-containing small molecule metabolic process				
nucleoside binding				
nucleoside diphosphate metabolic process				
nucleoside diphosphate phosphorylation				
nucleoside monophosphate metabolic process				
nucleoside phosphate binding				
nucleoside phosphate biosynthetic process				
nucleoside phosphate metabolic process				
nucleotide binding				
nucleotide biosynthetic process				
nucleotide metabolic process				
nucleotide phosphorylation				
Nucleotide-binding				
nucleotidyltransferase activity				
organic cyclic compound metabolic process				
organic substance biosynthetic process				
organonitrogen compound biosynthetic process				
organonitrogen compound metabolic process				
organophosphate biosynthetic process				
organophosphate metabolic process				
peptide biosynthetic process				
peptide metabolic process				
peptidoglycan-based cell wall biogenesis				
phosphate-containing compound metabolic process				
phosphorus metabolic process				
posttranscriptional regulation of gene expression				
Protein biosynthesis				
protein metabolic process				
purine nucleoside binding				
purine nucleoside diphosphate metabolic process				
purine nucleotide binding				
purine nucleotide metabolic process				
purine ribonucleoside binding				
purine ribonucleoside diphosphate metabolic process				
purine ribonucleoside triphosphate binding				
purine ribonucleotide binding				
purine ribonucleotide metabolic process				

Significant GO terms (p<=0.05) of L. gaesseri genes	Core	Soft-core	Shell	Cloud
purine-containing compound metabolic process				
Pyrimidine metabolism				
pyrimidine nucleotide biosynthetic process				
pyrimidine nucleotide metabolic process				
pyrimidine ribonucleotide biosynthetic process				
pyrimidine ribonucleotide metabolic process				
pyrimidine-containing compound biosynthetic process				
pyruvate metabolic process				
regulation of cellular amide metabolic process				
regulation of cellular protein metabolic process				
regulation of macromolecule metabolic process				
regulation of protein metabolic process				
regulation of translation				
response to extracellular stimulus				
ribonuclease activity				
ribonucleoprotein complex biogenesis				
Ribonucleoprotein, and Protein biosynthesis				
ribonucleoside binding				
ribonucleoside diphosphate metabolic process				
ribonucleoside monophosphate biosynthetic process				
ribonucleotide binding				
ribonucleotide biosynthetic process				
ribonucleotide metabolic process				
ribose phosphate biosynthetic process				
ribose phosphate metabolic process				
ribosomal subunit				
Ribosome				
ribosome biogenesis				
Ribosome, and Protein biosynthesis				
Ribosome, and RNA polymerase				
RNA binding				
RNA degradation				
RNA metabolic process				
RNA phosphodiester bond hydrolysis				
RNA phosphodiester bond hydrolysis, endonucleolytic				
RNA polymerase activity				
RNA processing				
RNA-binding				
rRNA binding				
S-adenosylmethionine-dependent methyltransferase activity				
SOS response				
transferase activity, transferring phosphorus-containing groups				
transition metal ion binding				
Translation				
translation factor activity, RNA binding				
translation regulator activity				
translation regulator activity, nucleic acid binding				
translational elongation				
tRNA aminoacylation				
tRNA aminoacylation for protein translation				
tRNA binding				
tRNA metabolic process				
tRNA processing				
zinc ion binding				
anatomical structure morphogenesis				
carbon-nitrogen ligase activity, with glutamine as amido-N-donor				
Cell cycle				
cell cycle process				

Significant GO terms (p<=0.05) of L. gaesseri genes	Core	Soft-core	Shell	Cloud
Cell division				
cell morphogenesis				
cytokinesis				
DNA-dependent ATPase activity				
glutaminyl-tRNA synthase (glutamine-hydrolyzing) activity				
glycosaminoglycan biosynthetic process				
glycosaminoglycan metabolic process				
helicase activity				
Homologous recombination				
intrinsic component of plasma membrane				
ligase activity, forming carbon-nitrogen bonds				
macromolecule methylation				
Methane metabolism				
monosaccharide metabolic process				
negative regulation of biological process				
negative regulation of cellular metabolic process				
negative regulation of cellular process				
negative regulation of gene expression				
negative regulation of macromolecule metabolic process				
negative regulation of metabolic process				
negative regulation of nitrogen compound metabolic process				
negative regulation of nucleobase-containing compound metabolic process				
organelle organization				
organic substance catabolic process				
peptidoglycan biosynthetic process				
peptidoglycan metabolic process				
regulation of anatomical structure morphogenesis				
regulation of cell morphogenesis				
regulation of cell shape				
regulation of developmental process				
3'-5' exonuclease activity				
amide transport				
ATPase activity				
Bacterial secretion system				
cellular amino acid metabolic process				
cellular macromolecule localization				
cellular protein localization				
cellular response to DNA damage stimulus				
cellular response to stress				
chromosome organization				
damaged DNA binding				
deoxyribonuclease activity				
DNA metabolic process				
DNA repair				
double-stranded DNA binding				
endodeoxyribonuclease activity				
endodeoxyribonuclease complex				
endonuclease activity				
endonuclease complex				
endopeptidase activity				
establishment of localization in cell				
establishment of protein localization				
excinuclease ABC activity				
excinuclease repair complex				
exonuclease activity				
galactose catabolic process				
galactose metabolic process				
hydrolase activity, acting on acid anhydrides				

Significant GO terms (p<=0.05) of L. gaesseri genes	Core	Soft-core	Shell	Cloud
hydrolase activity, acting on ester bonds				
intracellular protein transmembrane transport				
intracellular protein transport				
intracellular transport				
macromolecule catabolic process				
metalloendopeptidase activity				
metallopeptidase activity				
mismatch repair				
mismatched DNA binding				
nuclease activity				
nucleic acid phosphodiester bond hydrolysis				
nucleoside-triphosphatase activity				
Nucleotide excision repair				
nucleotide-excision repair				
peptidase activity				
peptide transport				
plasma membrane				
Protein export				
protein localization				
protein targeting				
protein transmembrane transport				
protein transport				
proteolysis				
pyrophosphatase activity				
Quorum sensing				
RNA modification				
tRNA modification				

Figure S3a: Significant functional enrichment analysis of the GOs using ClueGo, EnrichR, and ShinyGO search platform using the core, soft-core, shell, and cloud genes of *L. gasseri*

Lactobacillus gasseri

Significant GO terms (p<=0.05) of *L. gaesseri* genes	Core	Soft-core	Shell	Cloud
aromatic compound biosynthetic process				
ATP biosynthetic process				
ATP synthesis				
ATP synthesis coupled proton transport				
Carbon fixation in photosynthetic organisms				
catalytic activity, acting on a rRNA				
cation channel activity				
cation transmembrane transport				
cation transmembrane transporter activity				
cation transport				
cellular component disassembly				
cellular metabolic compound salvage				
cellular protein complex disassembly				
CF(1)				
channel activity				
CTP biosynthetic process				
CTP metabolic process				
endonuclease activity, active with either ribo- or deoxyribonucleic acids and producing 5'-phosphomonoesters				
energy coupled proton transport, down electrochemical gradient				
generation of precursor metabolites and energy				
glucose metabolic process				
heterocycle biosynthetic process				
Hydrogen ion transport				
Hydrogen ion transport, and DHHA2 domain				
inorganic cation transmembrane transport				
inorganic cation transmembrane transporter activity				
inorganic ion transmembrane transport				
inorganic molecular entity transmembrane transporter activity				
ion channel activity				
Ion transport				
membrane protein complex				
mixed, incl. Hydrogen ion transport, and DHHA2 domain				
monovalent inorganic cation transmembrane transporter activity				
monovalent inorganic cation transport				
N-methyltransferase activity				
nucleobase-containing compound kinase activity				
nucleoside monophosphate kinase activity				
nucleoside monophosphate phosphorylation				
nucleoside triphosphate biosynthetic process				
nucleoside triphosphate metabolic process				
nucleotide salvage				
organic cyclic compound biosynthetic process				
Oxidative phosphorylation				
passive transmembrane transporter activity				
phosphotransferase activity, phosphate group as acceptor				
Photosynthesis				
protein-containing complex disassembly				
protein-containing complex subunit organization				
proton channel activity				
proton transmembrane transport				
proton transmembrane transporter activity				
proton-transporting ATP synthase activity, rotational mechanism				
proton-transporting ATP synthase complex				
proton-transporting ATP synthase complex, catalytic core F(1)				
proton-transporting two-sector ATPase complex				
proton-transporting two-sector ATPase complex, catalytic domain				
purine nucleoside triphosphate biosynthetic process				
purine nucleoside triphosphate metabolic process				
purine nucleotide biosynthetic process				
purine ribonucleoside triphosphate biosynthetic process				
purine ribonucleoside triphosphate metabolic process				
purine ribonucleotide biosynthetic process				
purine-containing compound biosynthetic process				
pyrimidine nucleoside triphosphate biosynthetic process				
pyrimidine nucleoside triphosphate metabolic process				
pyrimidine ribonucleoside triphosphate biosynthetic process				
pyrimidine ribonucleoside triphosphate metabolic process				
pyrimidine-containing compound metabolic process				
regulation of biosynthetic process				
regulation of cellular biosynthetic process				
regulation of cellular macromolecule biosynthetic process				
regulation of cellular metabolic process				
regulation of macromolecule biosynthetic process				
Ribonucleoprotein				
ribonucleoside triphosphate biosynthetic process				
ribonucleoside triphosphate metabolic process				
Ribosomal protein				
rRNA metabolic process				
rRNA methylation				
rRNA methyltransferase activity				
rRNA modification				
rRNA processing				
rRNA-binding				
small ribosomal subunit				
translation elongation factor activity				
translation release factor activity				
translation termination factor activity				
translational termination				
unfolded protein binding				
Amino sugar and nucleotide sugar metabolism				
carbohydrate metabolic process				
Cell division, and CCB3/YggT				
Cell division, and D-Alanine metabolism				
cellular response to lipoteichoic acid				
cellular response to nicotine				
DNA recombination				
FtsZ-dependent cytokinesis				
galactose catabolic process				
Galactose metabolism				
glycoside metabolic process				
glycosyl compound catabolic process				
glycosylceramide catabolic process				
glycosylceramide metabolic process				
hexose metabolic process				
integral component of plasma membrane				
intra-Golgi vesicle-mediated transport				
intramolecular transferase activity				
Isomerase				
kinase activity				
Lipid biosynthesis				
Lipid metabolism				
methyltransferase activity				
mixed, incl. Cell division, and Penicillin-binding protein, transpeptidase				
monocarboxylic acid metabolic process				
Mur ligase family, catalytic domain				
Mur ligase, N-terminal catalytic domain				
NAD				
NAD(P)-binding domain				
negative regulation of biosynthetic process				
negative regulation of cellular biosynthetic process				
negative regulation of cellular macromolecule biosynthetic process				
negative regulation of macromolecule biosynthetic process				
negative regulation of nucleic acid-templated transcription				
negative regulation of RNA biosynthetic process				
negative regulation of RNA metabolic process				
negative regulation of transcription, DNA-templated				
One carbon pool by folate				
Phospholipid biosynthesis				
phospholipid biosynthetic process				
phospholipid metabolic process				
Phospholipid metabolism				
Phosphorylation				
regulation of miRNA metabolic process				
regulation of synaptic vesicle priming				
Repressor				
response to angiotensin				
response to lipoteichoic acid				
response to peptidoglycan				
RNA methylation				
RNA methyltransferase activity				
Transferase				
transferase activity, transferring one-carbon groups				
tRNA aminoacylation				
adenylyltransferase activity				
Alanine, aspartate and glutamate metabolism				
aminoacyl-tRNA editing activity				
aminoacyl-tRNA metabolism involved in translational fidelity				
Base excision repair				
Cell membrane				
cell septum assembly				
cellular catabolic process				
cellular macromolecule catabolic process				
Chromosome partition				
cytokinetic process				
'de novo' pyrimidine nucleobase biosynthetic process				
division septum assembly				
DNA biosynthetic process				
DNA damage				
DNA polymerase activity				
DNA-directed DNA polymerase activity				
DNA-templated transcription, termination				
Endonuclease				
establishment of protein localization to membrane				
GTP-binding				
Hydrolase				
integral component of membrane				
intrinsic component of membrane				
Magnesium				
Metal-binding				
mixed, incl. Homologous recombination, and Chaperone				
Nuclease				
nucleobase biosynthetic process				
nucleobase metabolic process				
nucleoside monophosphate biosynthetic process				
oxidoreductase activity				
pentose catabolic process				
pentose metabolic process				
pentose-phosphate shunt				
pentose-phosphate shunt, non-oxidative branch				
P-loop containing nucleoside triphosphate hydrolase				
protein localization to membrane				
pyrimidine deoxyribonucleotide catabolic process				
pyrimidine nucleobase biosynthetic process				
pyrimidine nucleobase metabolic process				
pyrimidine nucleoside monophosphate biosynthetic process				
pyrimidine nucleoside monophosphate metabolic process				
pyrimidine ribonucleoside monophosphate biosynthetic process				
pyrimidine ribonucleoside monophosphate metabolic process				
ribonucleoside monophosphate metabolic process				
SOS response, and KaiC				
transferase activity, transferring glycosyl groups				
transmembrane transport				
UMP biosynthetic process				
UMP metabolic process				
Zinc				
DNA binding				
DNA duplex unwinding				
DNA geometric change				
Glycine, serine and threonine metabolism				
macromolecule modification				
pentose catabolic process				
peptidase activity, acting on L-amino acid peptides				
regulation of metabolic process				

Figure S3b: Significant functional enrichment analysis of the GOs using ClueGo, EnrichR, and ShinyGO search platform using the core, soft-core, shell, and cloud genes of *L. gasseri*

Lactobacillus jensenni

**Significant GO terms (p<=0.05) of *L. jensenni* genes**	**Core**	**Soft-core**	**Shell**	**Cloud**
**amide biosynthetic process**	■	■		
**intracellular non-membrane-bounded organelle**	■	■		
**intracellular organelle**	■	■		
**mixed, incl. Ribosome, and Aminoacyl-tRNA biosynthesis**	■	■		
**mixed, incl. Ribosome, and Homologous recombination**	■	■		
**mixed, incl. Ribosome, and Pyrimidine metabolism**	■	■		
**mostly uncharacterized, incl. AAA domain (Cdc48 subfamily), and Methyltransferase small domain**	■	■		
**non-membrane-bounded organelle**	■	■		
**RNA binding**	■	■		
**rRNA binding**	■	■		
**ATPase activity**		■		■
**hydrolase activity, acting on acid anhydrides**		■		■
**hydrolase activity, acting on acid anhydrides, in phosphorus-containing anhydrides**		■		■
**nuclease activity**		■	■	
**nucleic acid phosphodiester bond hydrolysis**		■	■	
**nucleic acid phosphodiester bond hydrolysis**		■	■	
**nucleoside-triphosphatase activity**		■		■
**pyrophosphatase activity**		■		■
**ATP-binding**			■	■
**Cell cycle**			■	■
**Cell division**			■	■
**cellular response to DNA damage stimulus**			■	■
**cellular response to stress**			■	■
**Cytoplasm**			■	■
**DNA damage**			■	■
**DNA metabolic process**			■	■
**DNA repair**			■	■
**DNA replication**			■	■
**Homologous recombination, and DNA repair**			■	■
**Homologous recombination, and DNA replication**			■	■
**Homologous recombination, and SOS response**			■	■
**Isomerase**			■	■
**Ligase**			■	■
**Magnesium**			■	■
**Metal-binding**			■	■
**mixed, incl. Homologous recombination, and DNA repair**			■	■
**mixed, incl. Purine salvage, and GMP biosynthesis**			■	■
**Nucleotide-binding**			■	■
**P-loop containing nucleoside triphosphate hydrolase**			■	■
**tRNA processing**			■	■

**Significant GO terms (p<=0.05) of *L. jensenni* genes**	**Core**	**Soft-core**	**Shell**	**Cloud**
50S ribosome-binding GTPase	■			
amino acid activation	■			
Aminoacyl-tRNA biosynthesis	■			
aminoacyl-tRNA ligase activity	■			
Anticodon binding domain	■			
ATP biosynthetic process	■			
ATP synthesis coupled proton transport	■			
cation channel activity	■			
cation transmembrane transport	■			
cation transmembrane transporter activity	■			
cellular amide metabolic process	■			
cellular biosynthetic process	■			
cellular component disassembly	■			
cellular macromolecule biosynthetic process	■			
cellular macromolecule metabolic process	■			
cellular nitrogen compound biosynthetic process	■			
cellular nitrogen compound metabolic process	■			
cellular protein complex disassembly	■			
cellular protein metabolic process	■			
channel activity	■			
Elongation factor Tu GTP binding domain	■			
energy coupled proton transport, down electrochemical gradient	■			
gene expression	■			
inorganic cation transmembrane transport	■			
inorganic cation transmembrane transporter activity	■			
ion channel activity	■			
large ribosomal subunit	■			
ligase activity, forming carbon-oxygen bonds	■			
macromolecule biosynthetic process	■			
macromolecule metabolic process	■			
membrane protein complex	■			
mixed, incl. Amidase, and GatB domain	■			
mixed, incl. Oxidative phosphorylation, and Pyrimidine metabolism	■			
mixed, incl. Peptidoglycan biosynthesis, and YGGT family	■			
mixed, incl. Ribosomal protein L10, and Ribosomal L32p protein family	■			
mixed, incl. RNA polymerases D, and Ribosomal protein S8	■			
mixed, incl. Threonyl and Alanyl tRNA synthetase second additional domain, and B3/4 domain	■			
monovalent inorganic cation transmembrane transporter activity	■			
monovalent inorganic cation transport	■			
nucleic acid binding	■			
nucleobase-containing compound biosynthetic process	■			
organic substance biosynthetic process	■			
organonitrogen compound biosynthetic process	■			
organonitrogen compound metabolic process	■			
Oxidative phosphorylation	■			
Oxidative phosphorylation, and Haemolytic domain	■			
passive transmembrane transporter activity	■			
peptide biosynthetic process	■			
peptide metabolic process	■			
phosphotransferase activity, phosphate group as acceptor	■			
posttranscriptional regulation of gene expression	■			
protein metabolic process	■			
protein-containing complex disassembly	■			
protein-containing complex subunit organization	■			

**Significant GO terms (p<=0.05) of *L. jensenni* genes**	**Core**	**Soft-core**	**Shell**	**Cloud**
proton channel activity	■			
proton transmembrane transport	■			
proton transmembrane transporter activity	■			
proton-transporting ATP synthase activity, rotational mechanism	■			
proton-transporting ATP synthase complex	■			
proton-transporting ATP synthase complex, catalytic core F(1)	■			
proton-transporting two-sector ATPase complex	■			
proton-transporting two-sector ATPase complex, catalytic domain	■			
purine nucleoside triphosphate biosynthetic process	■			
purine nucleoside triphosphate metabolic process	■			
purine ribonucleoside triphosphate biosynthetic process	■			
purine ribonucleoside triphosphate metabolic process	■			
purine-containing compound salvage	■			
regulation of biosynthetic process	■			
regulation of cellular amide metabolic process	■			
regulation of cellular biosynthetic process	■			
regulation of cellular component organization	■			
regulation of cellular macromolecule biosynthetic process	■			
regulation of cellular metabolic process	■			
regulation of cellular process	■			
regulation of cellular protein metabolic process	■			
regulation of gene expression	■			
regulation of macromolecule biosynthetic process	■			
regulation of macromolecule metabolic process	■			
regulation of metabolic process	■			
regulation of nitrogen compound metabolic process	■			
regulation of primary metabolic process	■			
regulation of protein metabolic process	■			
regulation of protein-containing complex disassembly	■			
regulation of translation	■			
ribonucleoprotein complex binding	■			
ribonucleoside triphosphate biosynthetic process	■			
ribonucleoside triphosphate metabolic process	■			
ribosomal subunit	■			
Ribosome	■			
ribosome binding	■			
Ribosome, and Aminoacyl-tRNA biosynthesis	■			
Ribosome, and Bacterial secretion system	■			
RNA metabolic process	■			
small ribosomal subunit	■			
Translation	■			
translation elongation factor activity	■			
translation factor activity, RNA binding	■			
translation regulator activity	■			
translation regulator activity, nucleic acid binding	■			
translation release factor activity	■			
translation termination factor activity	■			
translational elongation	■			
translational termination	■			
tRNA aminoacylation	■			
tRNA aminoacylation for protein translation	■			
tRNA binding	■			
tRNA synthetase class II core domain (G, H, P, S and T)	■			
tRNA synthetases class II (D, K and N)	■			
Aspartate-ammonia ligase		■		
bone resorption		■		
cAMP metabolic process		■		
Chaperonin 10 Kd subunit		■		
CoA binding domain		■		

**Significant GO terms (p<=0.05) of *L. jensenni* genes**	**Core**	**Soft-core**	**Shell**	**Cloud**
Domain of unknown function (DUF3552)		■		
GrpE		■		
hydrolase activity, acting on ester bonds		■		
KH domain		■		
Leucyl-tRNA synthetase, Domain 2		■		
macromolecule biosynthetic process		■		
mixed, incl. AAA domain (Cdc48 subfamily), and RNA degradation		■		
mixed, incl. GrpE, and Chaperonin 10 Kd subunit		■		
mixed, incl. RNB domain, and SmpB protein		■		
ncRNA metabolic process		■		
Peptidyl-tRNA hydrolase		■		
positive regulation of alcohol biosynthetic process		■		
positive regulation of cellular carbohydrate metabolic process		■		
positive regulation of glycogen biosynthetic process		■		
positive regulation of glycogen metabolic process		■		
positive regulation of inositol phosphate biosynthetic process		■		
PPIC-type PPIASE domain		■		
Putative DNA-binding protein N-terminus		■		
RecG wedge domain		■		
regulation of inositol phosphate biosynthetic process		■		
ribonuclease activity		■		
Ribonuclease B OB domain		■		
Ribosomal protein S15		■		
Ribosomal protein S20		■		
Ribosomal RNA adenine dimethylases		■		
RNA degradation		■		
RNA phosphodiester bond hydrolysis		■		
RNB domain		■		
RsgA N-terminal domain		■		
TCP-1/cpn60 chaperonin family		■		
Type I 3-dehydroquinase		■		
cell cycle process			■	
Cell membrane			■	
cell septum assembly			■	
cellular metabolic compound salvage			■	
coenzyme A biosynthetic process			■	
coenzyme A metabolic process			■	
CTP biosynthetic process			■	
CTP metabolic process			■	
Cytokinesis			■	
cytokinetic process			■	
division septum assembly			■	
FtsZ-dependent cytokinesis			■	
galactose catabolic process			■	
galactose metabolic process			■	
galactose metabolic process			■	
hexose catabolic process			■	
Hydrolase			■	
hydrolase activity, hydrolyzing N-glycosyl compounds			■	
integral component of plasma membrane			■	
intrinsic component of plasma membrane			■	
Kinase			■	
macromolecule modification			■	
mixed, incl. Purine biosynthesis, and Pyrimidine biosynthesis			■	
mixed, incl. Ribokinase, and D-ribose pyranase			■	
mixed, incl. SLBB domain, and Competence protein			■	
mixed, incl. SLBB domain, and Impact family			■	
ncRNA processing			■	
Nuclease			■	

**Significant GO terms (p<=0.05) of *L. jensenni* genes**	**Core**	**Soft-core**	**Shell**	**Cloud**
nucleic acid metabolic process			■	
nucleobase-containing compound kinase activity			■	
nucleobase-containing compound metabolic process			■	
nucleoside bisphosphate biosynthetic process			■	
nucleoside monophosphate kinase activity			■	
nucleoside monophosphate phosphorylation			■	
nucleoside triphosphate biosynthetic process			■	
nucleotide-excision repair			■	
one-carbon metabolic process			■	
plasma membrane			■	
Purine biosynthesis, and Pyrimidine biosynthesis			■	
purine nucleoside bisphosphate biosynthetic process			■	
purine nucleoside metabolic process			■	
purine ribonucleoside metabolic process			■	
Pyrimidine metabolism, and Purine biosynthesis			■	
pyrimidine nucleoside triphosphate biosynthetic process			■	
pyrimidine nucleoside triphosphate metabolic process			■	
pyrimidine ribonucleoside triphosphate biosynthetic process			■	
pyrimidine ribonucleoside triphosphate metabolic process			■	
ribonucleoside bisphosphate biosynthetic process			■	
ribonucleoside metabolic process			■	
RNA modification			■	
RNA processing			■	
small molecule catabolic process			■	
Transferase			■	
tRNA modification			■	
ATP synthesis				■
Cell shape				■
cell wall biogenesis				■
Cell wall biogenesis/degradation				■
cell wall macromolecule biosynthetic process				■
cell wall macromolecule metabolic process				■
cellular catabolic process				■
cellular component macromolecule biosynthetic process				■
cellular component organization				■
cellular response to external stimulus				■
cellular response to extracellular stimulus				■
Chaperone, and ClpP, Ser active site				■
chromosome				■
chromosome organization				■
ClpA/B family				■
DNA binding				■
DNA conformation change				■
DNA-dependent ATPase activity				■
DNA-dependent DNA replication				■
glutaminyl-tRNA synthase (glutamine-hydrolyzing) activity				■
Homologous recombination, and PD-(D/E)XK nuclease superfamily				■
Hydrogen ion transport				■
Ion transport				■
negative regulation of macromolecule metabolic process				■
organelle organization				■
Peptidoglycan synthesis				■
peptidoglycan-based cell wall biogenesis				■
Repeat				■
response to extracellular stimulus				■
SOS response				■

Figure S4: Significant functional enrichment analysis of the GOs using ClueGo, EnrichR, and ShinyGO search platform using the core, soft-core, shell, and cloud genes of *L. jensenii*
