# Supplementary material for: Comparative pangenomic analysis of predominant human vaginal lactobacilli strains towards population-specific adaptation: understanding the role in sustaining a balanced and healthy vaginal microenvironment
Source: BMC Genomics. 2023 Sep 22;24:565. doi: 10.1186/s12864-023-09665-y (PMC10517566; doi:10.1186/s12864-023-09665-y)
Supplement: Supplementary file 3 — Supplementary Material 3 [file 12864_2023_9665_MOESM3_ESM.docx]

**Additional file 3: Table S2:** Key attributes-related parent and child GO terms, associated genes, and the relevant literature that relates the GO term with the key functionalities.

| Key functions | GO terms involved (parent, child, daughter) | Associated genes | Literature associating the biological process/molecular function with the key function |
| --- | --- | --- | --- |
| Plasma membrane integrity | FAB  (GO:0006633, GO:0000037  Category:  Biological Process)  Parent GO: GO:0006631) | *acc*B, *acc*C, *acc*D, *acp*P, *fab*H, *fab*I, *fab*Z | [1, 2] |
| Adherence, aggregation, and exopolysaccharide synthesis | CDB  (GO:0097367,  Category: Molecular Function,  parent GO:0005488) | *ack*A, *atp*A, *atp*F, *car*B, *coa*D, *dac*A, *dlt*A, *glm*S, *gm*k, *gpm*A, *gps*A, *mra*Y, *mur*C, *mur*D, *mur*F, *mur*I, *pgk*, *pyr*B, *pyr*C, *pyr*D, *rbs*K, *rpi*A | [3, 4] |
|  | PB  (GO:0005515, GO:0001948, GO:0045308)  Category: Molecular Function  Parent GO: GO:0005488) | *dna*J, *dna*K, *gro*L, *hsl*O | [5-7] |
|  | PTTA  (GO:0022803, GO:0022814)  Category: Molecular Function  Parent GO: GO:0022857) | *atp*A, *atp*B, *atp*C, *atp*D, *atp*F, *atp*G, *atp*H | [8] |
|  | TA  (GO:0005215, GO:0005478)  Category: Molecular Function  Parent GO: GO:0003674) | *atp*A, *atp*B, *atp*C, *atp*D, *atp*F, *atp*G, *atp*H, *kup* | [8] |
|  | GM  (GO:0006012)  Category: Biological Process  Parent GO: GO:0019318) | *gal*M, *gal*T, *gal*K, *lac*Z, *lac*R, *lac*L, *lac*M | [9, 10] |
|  | CA  (GO:0006457, GO:0007022, GO:0007024, GO:0007025)  Category: Biological Process  Parent GO: GO:0009987,  GO:0008151, GO:0044763, GO:0050875, GO:0051604) | *prm*A, *clp*C, *pth, mfd*, *clp*B, *grp*E, *hrc*A, *clp*E, *clp*P | [11, 12] |
|  | AgBP  (GO:0006023)  Category: Biological Process  Parent GO: GO:0006022) | *mra*Y, *mur*C, *mur*D, *mur*F, *mur*I | [13-15] |
|  | AFABP  (GO:0009067)  Category: Biological Process  Parent GO: GO:0009066, GO:1901607) | *asn*A, *dap*F, *dap*H, *lys*A, *mtn*N, *thr*B, *asn*S | [16, 17] |
|  | GaBP  (GO:0006024)  Category: Biological Process  Parent GO: GO:0006023, GO:0030203) | *mra*Y, *mur*C, *mur*D, *mur*F, *mur*I, *mur*B, *mur*G | [13] |
| Biosurfactant synthesis | Hydrolase transferase  (GO:0016817)  Category: Molecular Function  Parent: GO:0016787 | *pcp*, *pepd*A, *muts*2, *rdg*B, *clp*P, *cbh*, *rib*D, *rec*Q,*ppa*C, *din*G, *add*A, *cas*2, *arc*A, *lep*A, *lex*A, *rec*G, *xse*A, *folD*, *lac*Z, *lac*L, *lac*M, *mut*M, *pol*A *pep*N, *csh*B, *ffh*, *fts*H, *fts*Y, *gyr*A, *gyr*B, *inf*B, *mut*L, parC, *par*E, *prf*C, *rex*B, *rsg*A, *ruv*B | [18, 19] |
|  | GCMP  (GO:1901657)  Category: Biological Process  Parent GO  : GO:1901135 | *apt*, *gua*A, *prs*, *pyr*R | [20, 21] |
|  | GcMP  (GO:0006677)  Category: Biological Process  Parent GO: GO:0006687, GO:0006672 | *gla* | [22] |
|  | SM  (GO:0006665)  Category: Biological Process  Parent GO: GO:0006643 | *gal*M, *lac*Z, *lac*L, *lac*M | [23] |
|  | CMBP  (GO:0044038)  Category: Category: Biological Process  Parent GO: GO:0042546 | *ala*S, *asp*S, *dlt*A, *dlt*C, *dna*A, *dna*G, *dna*J, *efp*, *frr*, *fus*A, *glm*U, *glt*X, *gre*A, *gyr*B, *his*S, *ile*S, *inf*A, *lys*S, *met*G, murE, *mur*I, *nus*A, *phe*S, *pol*C, *prf*A, *prf*B, *prf*C, *pth*, *pyr*R, *rec*F, *rpl*B, *rpl*C, *rpl*D, *rpl*E, *rpl*F, *rpl*I, *rpl*O, *rpl*P, *rpl*Q, *rpl*R, *rpl*V, *rpl*W, *rpl*X, *rpm*C, *rpm*D, *rpm*J, *rpo*A, *rpo*E, *rps*B, *rps*C, *rps*D, *rps*E, *rps*F, *rps*G, *rps*H, rpsJ, rpsK, *rps*L, *rps*M, *rps*Q, *rps*R, *rps*S, *rsf*S, *sig*A, *smc*, *tuf*, *tyr*S, *val*S | [24] |
| Bacteriocin synthesis | ABCT  (GO:0140359)  Category: Molecular Function  Parent GO: GO:0042626, GO:0015405, GO:0016820, GO:0043492 | *ytr*E, *ecs*A, *yhe*S, *nat*A, *ydc*V,  *gln*H, *gln*M, *gln*P, *psa*A, *lag*D, *ybi*T, *ssa*B, *art*L, *yhe*H, *yhe*L, *ydc*B, *phn*D,  *btu*D, *lmr*A, *yxd*L, *fim*A, *nos*F | [25, 26] |
|  | TTA  (GO:0008324)  Category: Molecular Function  Parent GO: GO:0015075 | *atp*A, *atp*B, *atp*C, *atp*D, *atp*F, *atp*G, *atp*H | [27, 28] |
|  | *Nis* | *nis*B*,nis*C | [29] |
| Hydrogen peroxide and organic acid biosynthesis  D/L-lactate metabolic processes | *Pox* | Pyruvate oxidase *(pox)* | [30] |
|  | *Nox* | NADH oxidase *(nox)* | [31] |
|  | OSMP  (GO:0071704)  Category: Biological Process  Parent GO: GO:0008152, GO:0044236, GO:0044710,  GO:0034641,  GO:0044237) | *ack*A, *acp*P, *acp*S, *adk*, *ala*S, *alr*, *asd*, *asn*B, *asp*S, *atp*A, *atp*B, *atp*C, *atp*D, *atp*H, *car*A, *car*B, *cmk*, *coa*D, *cys*S, *dac*A, *dap*A, *dap*B, *dap*H, *ddl*A, *def*, *din*G, *dlt*A, *dlt*C, *dna*A, *dna*G, *dna*J, *efp*, *frr*, *gat*A, *gat*C, *glm*U, *gln*A, *glt*X, *gly*A, *gly*Q, *gly*S, *gmk*, *gpm*A, *gps*A, *gre*A, *gyr*A, *gyr*B, *his*S, *hpt*, *hrc*A, *inf*A, *inf*B, *inf*C, *lex*A, *lgt*, *lig*A, *met*G, *met*K, *mfd*, *mra*Z, *mtn*N, *mur*B, *mur*C, *mur*D, *mur*F, *mur*G, *nad*D, *nad*E, *nus*A, *nus*B, *nus*G, *pcr*A, *pgi*, *pgs*A, *phe*S, *phe*T, *pls*X, *pls*Y, *pol*C, *prf*A, *prf*B, *prf*C, *pro*S, *prs*, *pur*A, *pyr*B, *pyr*C, *pyr*DB, *pyr*E, *pyr*F, *pyr*G, *pyr*H, *pyr*R, *rec*F, *rpl*A, *rpl*B, *rpl*C, *rpl*D, *rpl*E, *rpl*F, *rpl*I, *rpl*J, *rpl*K, *rpl*L, *rpl*M, *rpl*N, *rpl*O, *rpl*P, *rpl*R, *rpl*S, *rpl*T, *rpl*U, *rpl*V, *rpl*W, *rpl*X, *rpm*A, *rpm*B, *rpm*C, *rpm*D, *rpm*F, *rpm*I, *rpo*A, *rpo*B, *rpo*E, *rpo*Z, *rps*B, *rps*C, *rps*D, *rps*E, *rps*F, *rps*G, *rps*H, *rps*J, *rps*K, *rps*L, *rps*M, *rps*O, *rps*P, *rps*Q, *rps*R, *rps*S, *rps*T, *rsf*S, *scp*A, *scp*B, *ser*S, *smp*B, *tdk*, *thi*I, *thy*A, *tmk*, *tpi*A, *tsf*, *tuf*, *upp*, *val*S, *wal*R, *xpt*, *yab*A.*ldh* | [32-34] |

- FAB – Fatty acid biosynthesis; CDB – Carbohydrate derivative binding; PB – Protein binding; PTTA – passive transmembrane transporter activity; TA – Transporter activity; GM - Galactose metabolism; CA - Chaperone activity; AgBP- Aminoglycan biosynthetic process; AFABP - Aspartate family amino acid biosynthetic process; GaBP - Glycosaminoglycan biosynthetic process ABCT – ABC transporter; TTA – Transmembrane transporter activity; *Nis*– *Nisin* *synthase*; *Pox* – *Pyruvate oxidase*; *Nox* – *NADH oxidase*; OSMP – Organic substance metabolic process; GCMP – Glycosyl compound metabolic process; GcMP - Glycosylceramide metabolic process; SM - Sphingolipid metabolism; CMBP – Cellular macromolecule biosynthetic process.
